# Supplementary material for: Mesoporous Pt@Pt-skin Pt3Ni core-shell framework nanowire electrocatalyst for efficient oxygen reduction
Source: Nat Commun. 2023 Mar 18;14:1518. doi: 10.1038/s41467-023-37268-4 (PMC10024750; doi:10.1038/s41467-023-37268-4)
Supplement: Supplementary file 1 — Supplementary Information [file 41467_2023_37268_MOESM1_ESM.docx]

Supplementary Materials for

**Mesoporous Pt@Pt-skin Pt_3_Ni core-shell framework nanowire electrocatalyst for efficient oxygen reduction**

Hui Jin^1†^, Zhewei Xu^1†^, Zhi-Yi Hu^1†^, Zhiwen Yin^1^, Zhao Wang^1^, Zhao Deng^1^, Ping Wei^1^, Shihao Feng^1^, Shunhong Dong^1^, Jinfeng Liu^1^, Sicheng Luo^1^, Zhaodong Qiu^1^, Liang Zhou^1^, Liqiang Mai^1^, Bao-Lian Su^1,2^, Dongyuan Zhao^3^ and Yong Liu^1*^

^1^ International School of Materials Science and Engineering (ISMSE), State Key Laboratory of Advanced Technology for Materials Synthesis and Processing, Wuhan University of Technology, Wuhan 430070, China.

^2^ Laboratory of Inorganic Materials Chemistry, Department of Chemistry, University of Namur, 61 rue de Bruxelles, B-5000 Namur, Belgium

^3^ Department of Chemistry, Laboratory of Advanced Materials, Shanghai Key Lab of Molecular Catalysis and Innovative Materials, State Key Laboratory of Molecular Engineering of Polymers, Fudan University, Shanghai 200433, P.R. China.

*Corresponding author. E-mail: [liuyong3873@whut.edu.cn](mailto:liuyong3873@whut.edu.cn)

†These authors contributed equally to this work

This PDF file includes:

Supplementary Figure 1 to 31

Supplementary Table 1 to 6

Supplementary Reference 1 to 9


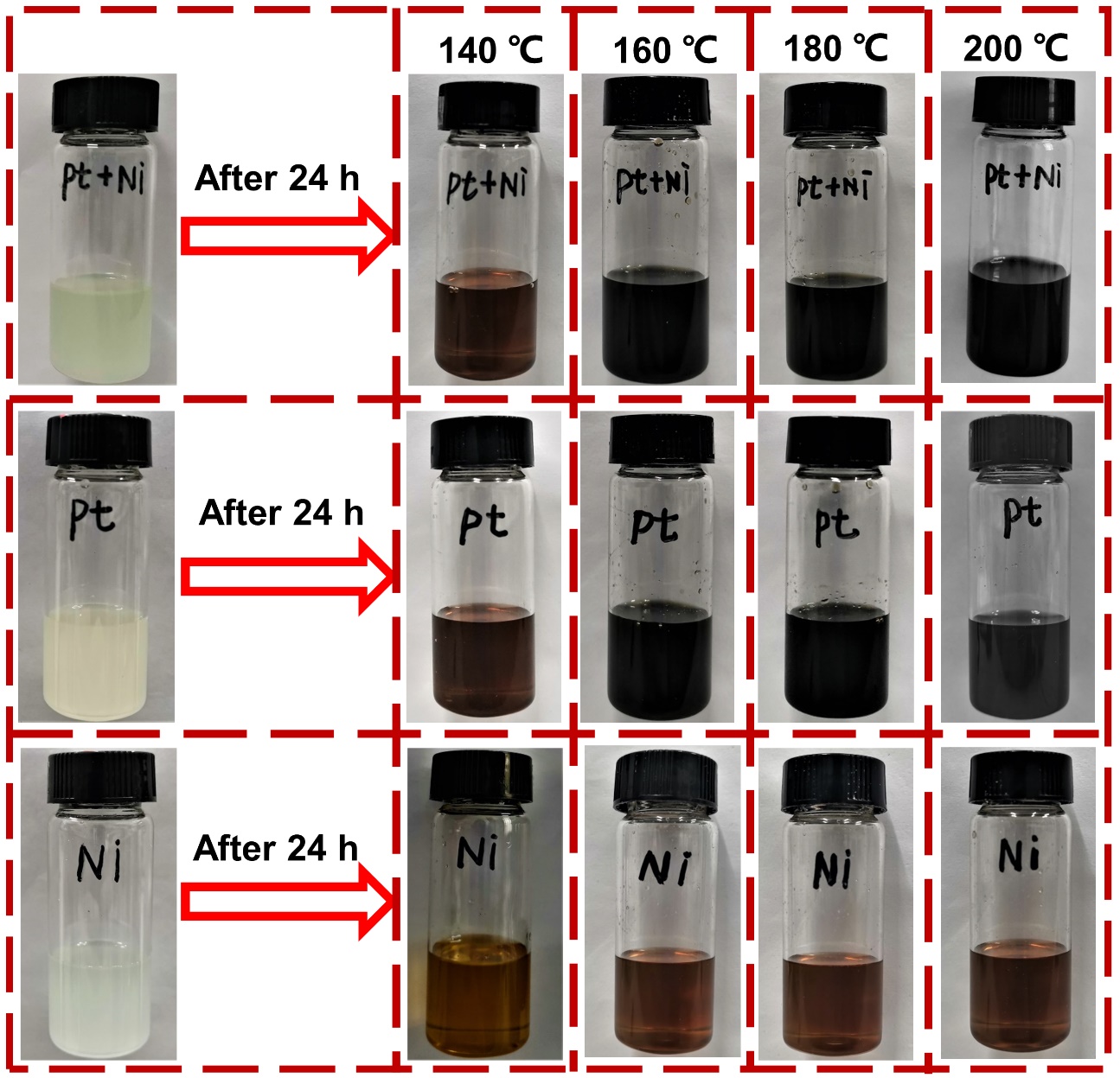
**Supplementary Figure 1.** Photographs of the Pt/Ni precursor solution before and after reaction. The Pt(acac)_2_ and Ni(acac)_2_ reduction ability comparison experiment results show that the reduction of Pt(acac)_2_/Ni(acac)_2_ mixture and pure Pt(acac)_2_ begins at approximately 160°C. However, the pure Ni(acac)_2_ (without Pt^2+^ precursor) cannot be reduced even under high reaction temperature of 200°C, profoundly demonstrating that Pt^2+^ ions have more positive reduction potential than that of Ni^2+^ ions under the same reaction conditions and the initially reduced Pt has a catalytic effect on the reduction of Ni.


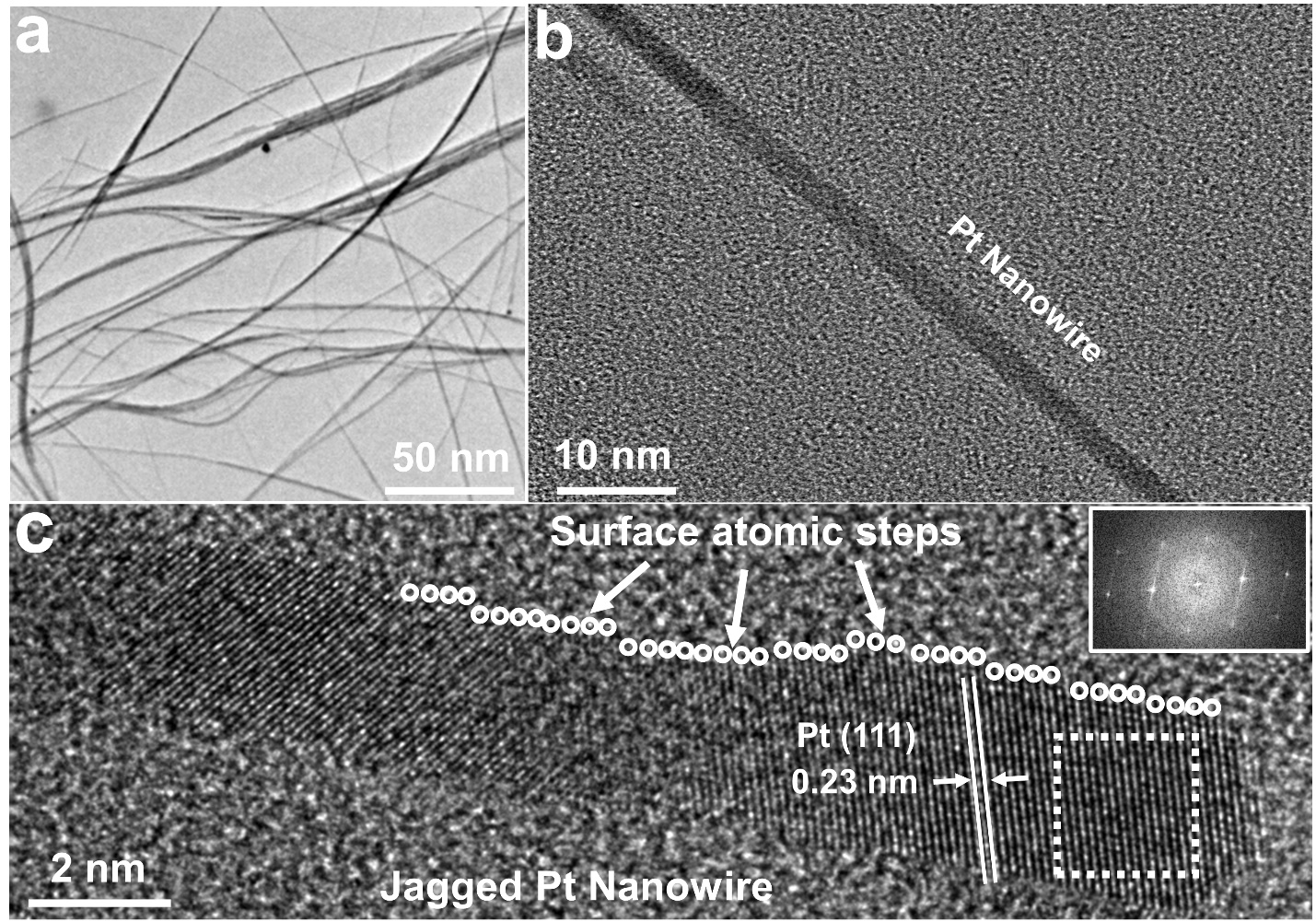
**Supplementary Figure 2. (a, b)** Representative TEM images, **(c)** HRTEM image of interim atomic-jagged Pt nanowires collected after a growth period of 30 min.


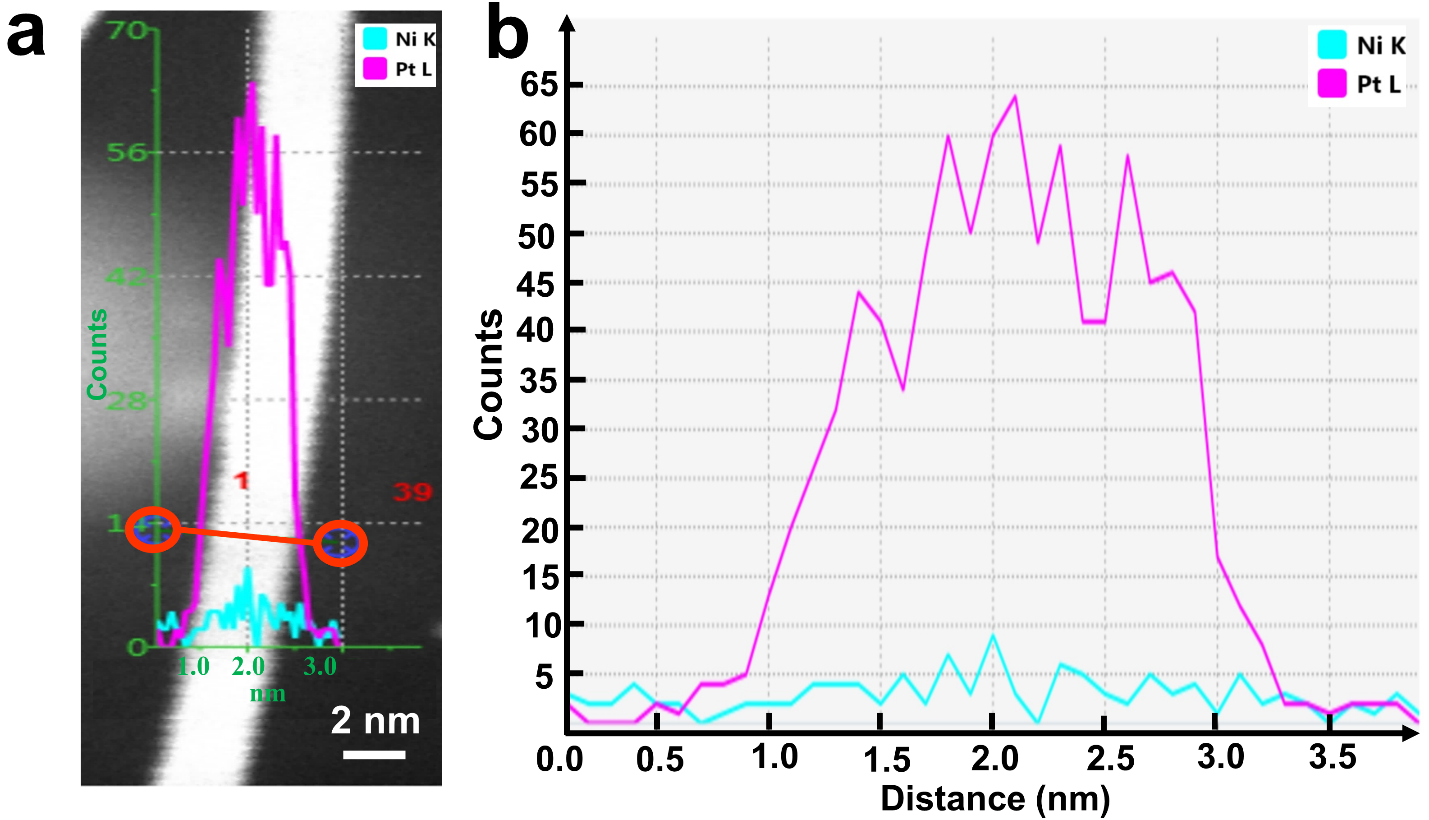
**Supplementary Figure 3.** (**a**) STEM image of initial atomic-jagged Pt nanowires and (**b**) the corresponding STEM-EDX line-scanning profile along the red line.


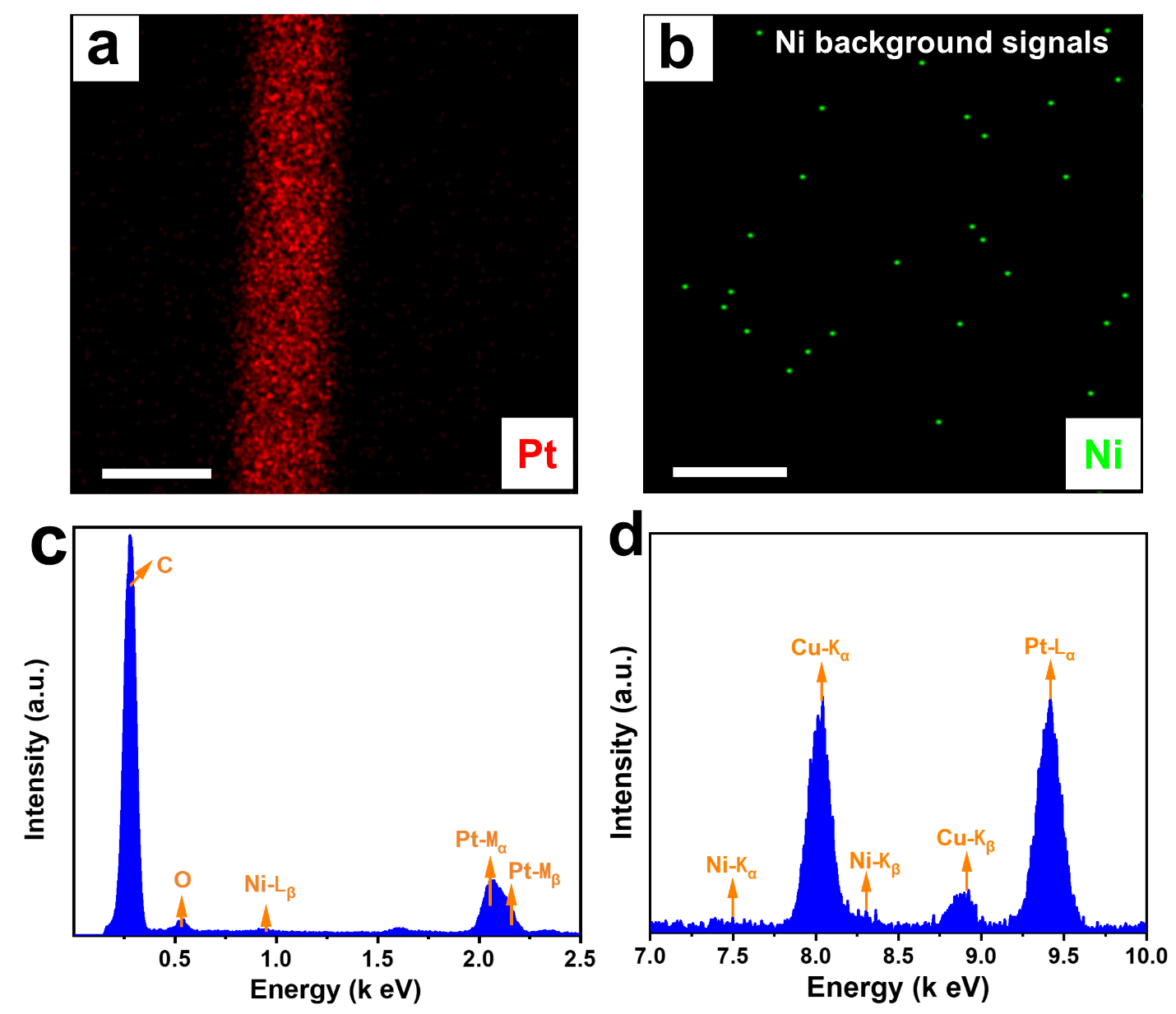
**Supplementary Figure 4.** (**a-b**) EDS elemental mapping of initially formed atomic-jagged Pt nanowire. scale bars in (a) to (b), 3 nm. **(c-d)** Corresponding EDS intensity images. As shown in Supplementary Figure 4a-c, Pt is the only element in the nanowires, while Ni only exists as background noise. In addition, the EDS intensity images illustrate that all Ni element peaks (Ni-K_α_, Ni-K_β_, Ni-L_β_ peaks) are the background noise. Consequently, we consider that there is no Ni element in the initially formed nanowires.


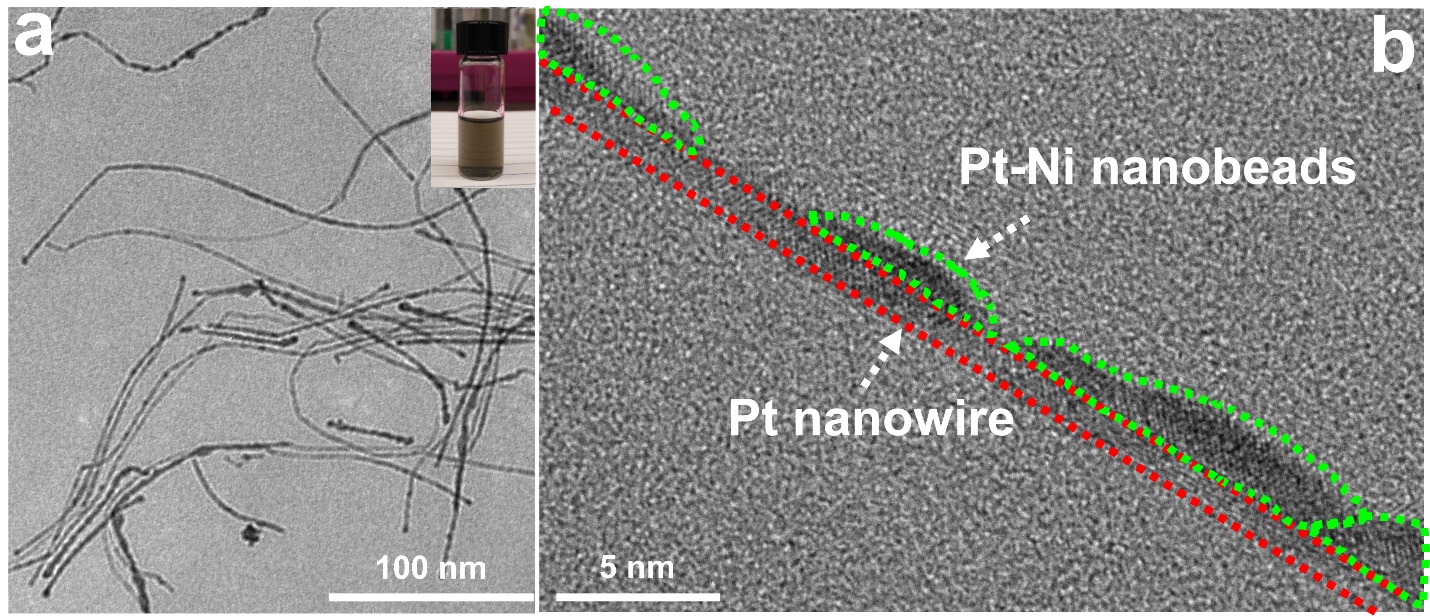
**Supplementary Figure 5.** (**a**) TEM image of Pt@PtNi nanowire intermediates. The inset is photograph of the solution collected after a reaction period of 2 hours. (**b**) HRTEM image of Pt@PtNi nanowire intermediates. It is found that Pt-Ni alloy phase selectively deposits on the defective surface sites of Pt nanowires.


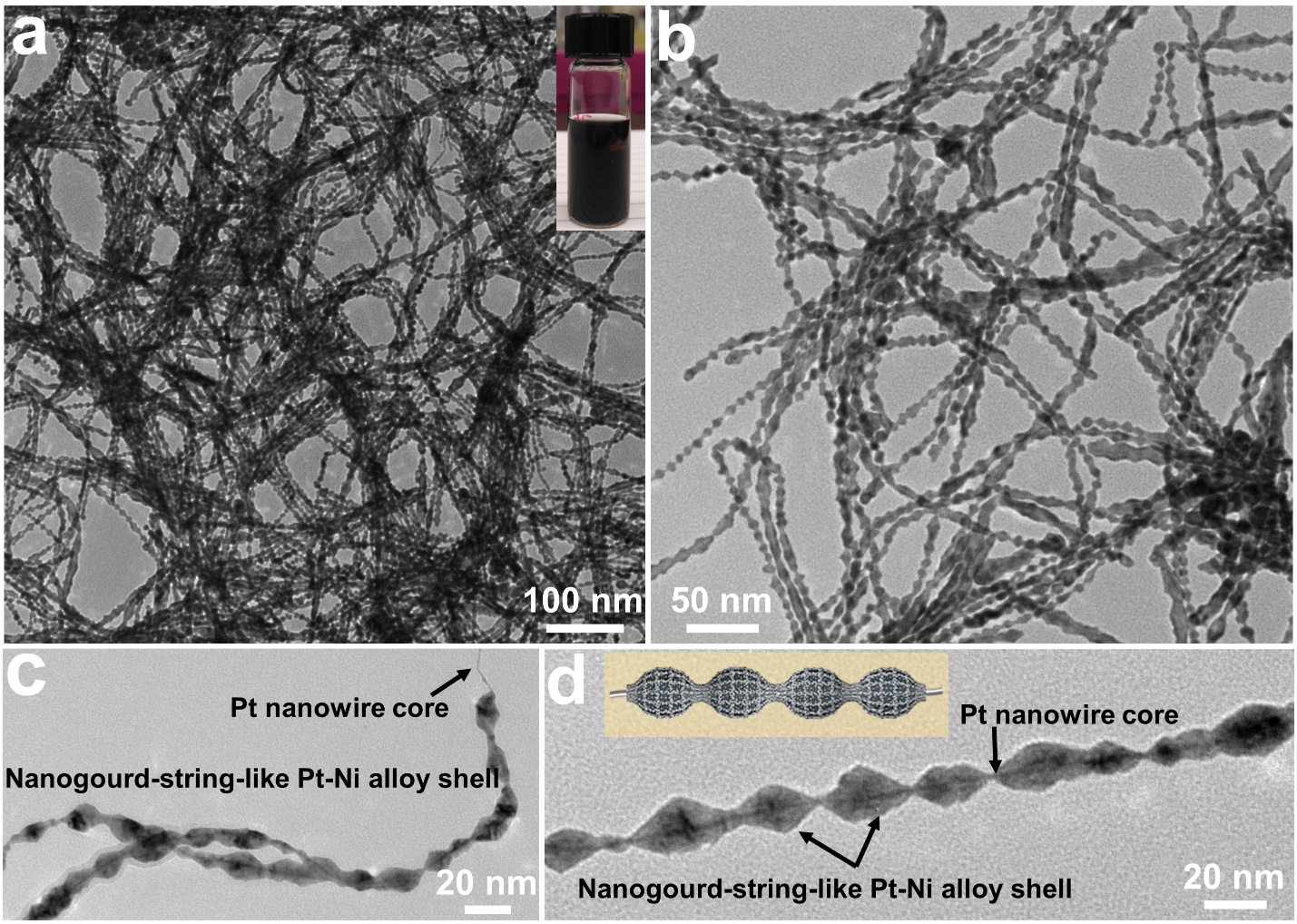
**Supplementary Figure 6. (a-d)** Typical TEM images of Pt@Pt-Ni alloy core-shell nanowires (CSNWs). The inset in **(a)** is photograph of the solution collected after a reaction period of 24 hours. The results indicate the formation of uniform nanogourd-string-like Pt@Pt-Ni alloy CSNWs.


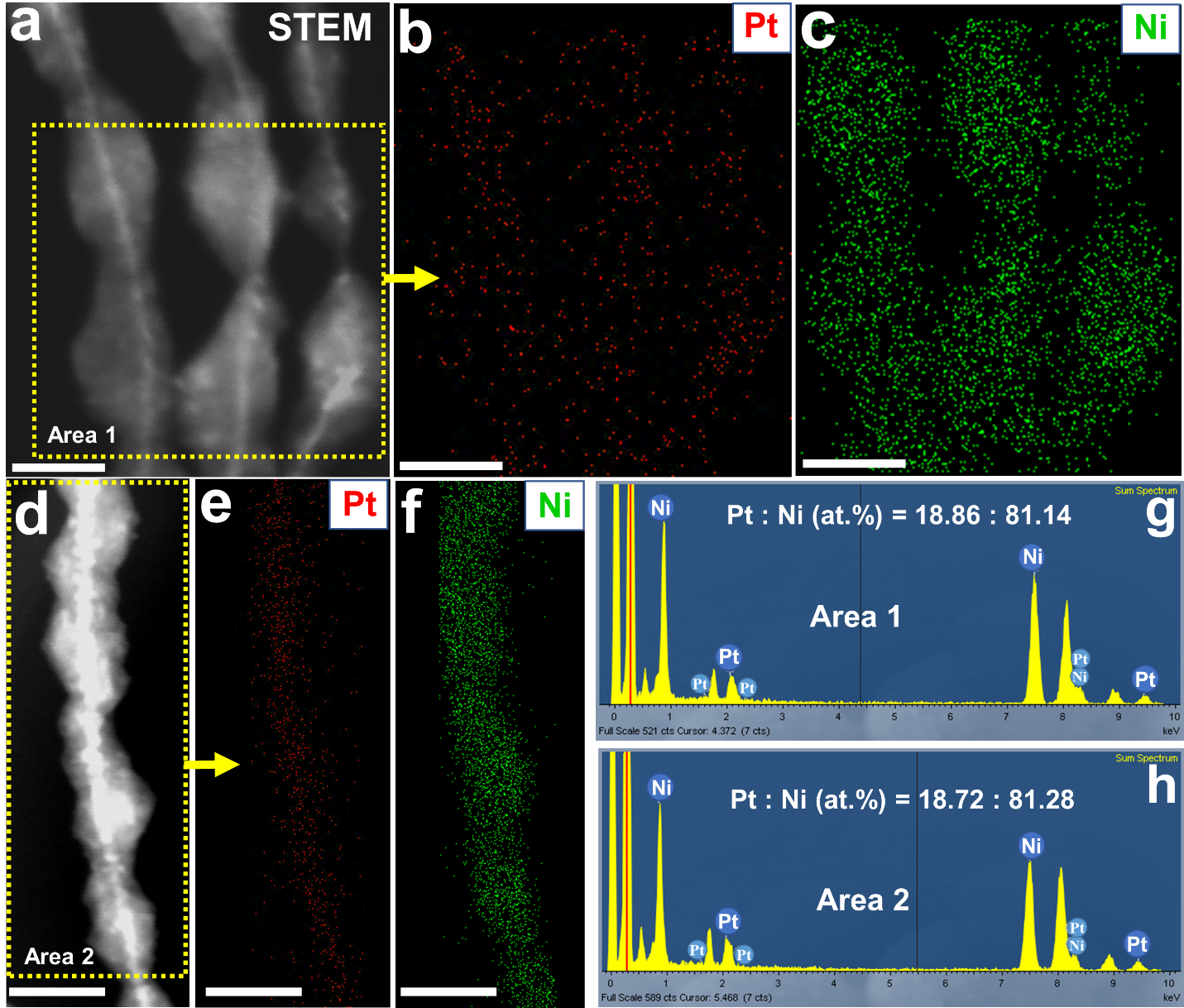
**Supplementary Figure 7. (a**, **d**) HAADF-STEM images of Pt@Pt-Ni alloy CSNWs and (**b**, **c**, **e**, **f**) the corresponding EDS element mapping images of the areas marked by the yellow dashed squares, Pt (red) and Ni (green). scale bars in (a) to (f), 20 nm. (**g**, **h**) TEM-EDS data of two different areas in **(a)** and **(d)**, indicating that the overall Pt /Ni atomic ratio is approximately 1:4.3.


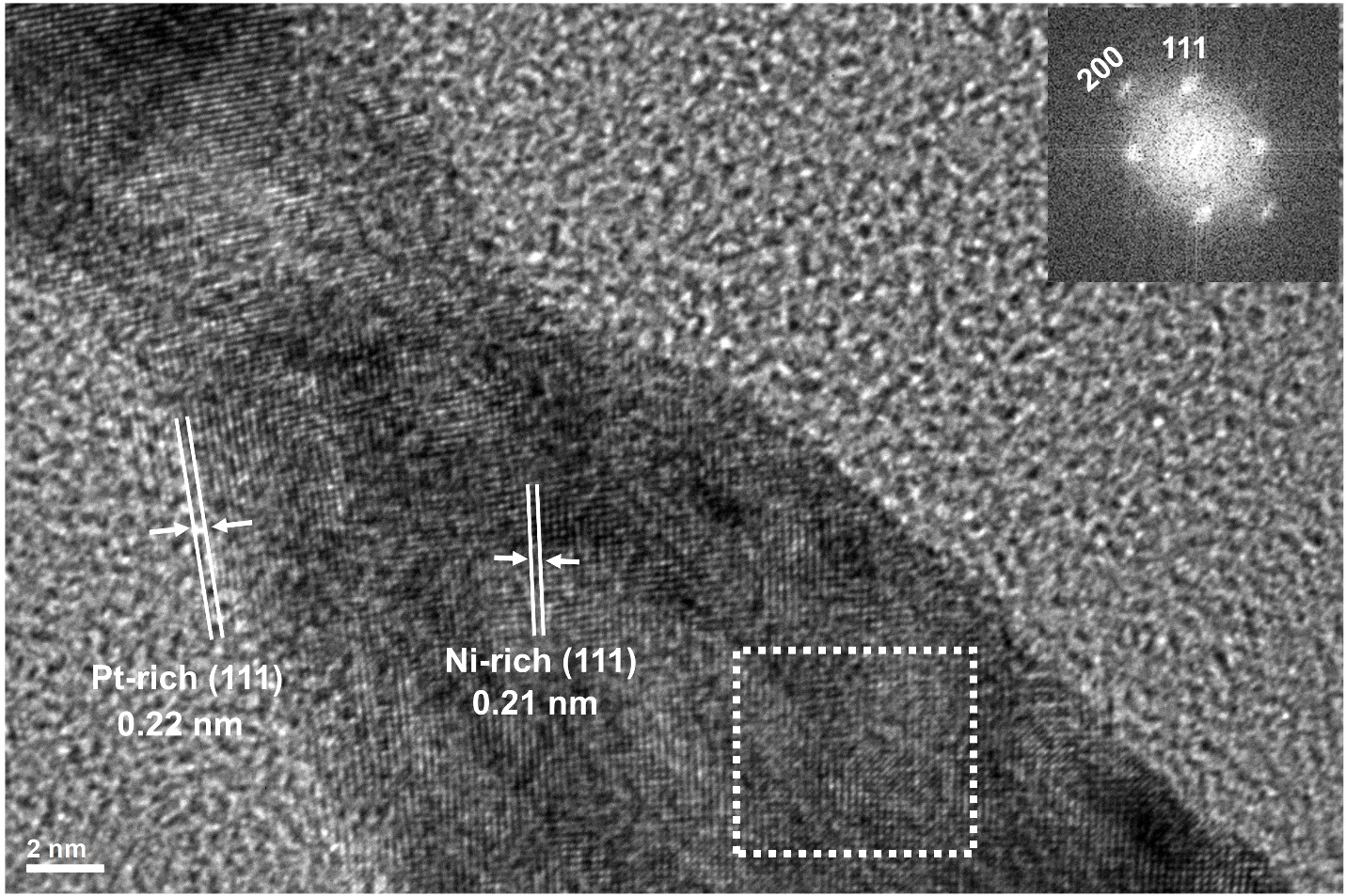
**Supplementary Figure 8.** HRTEM image of one single Pt@Pt-Ni CSNWs. The inset is the FFT pattern from the white dashed square inside the nanowire.


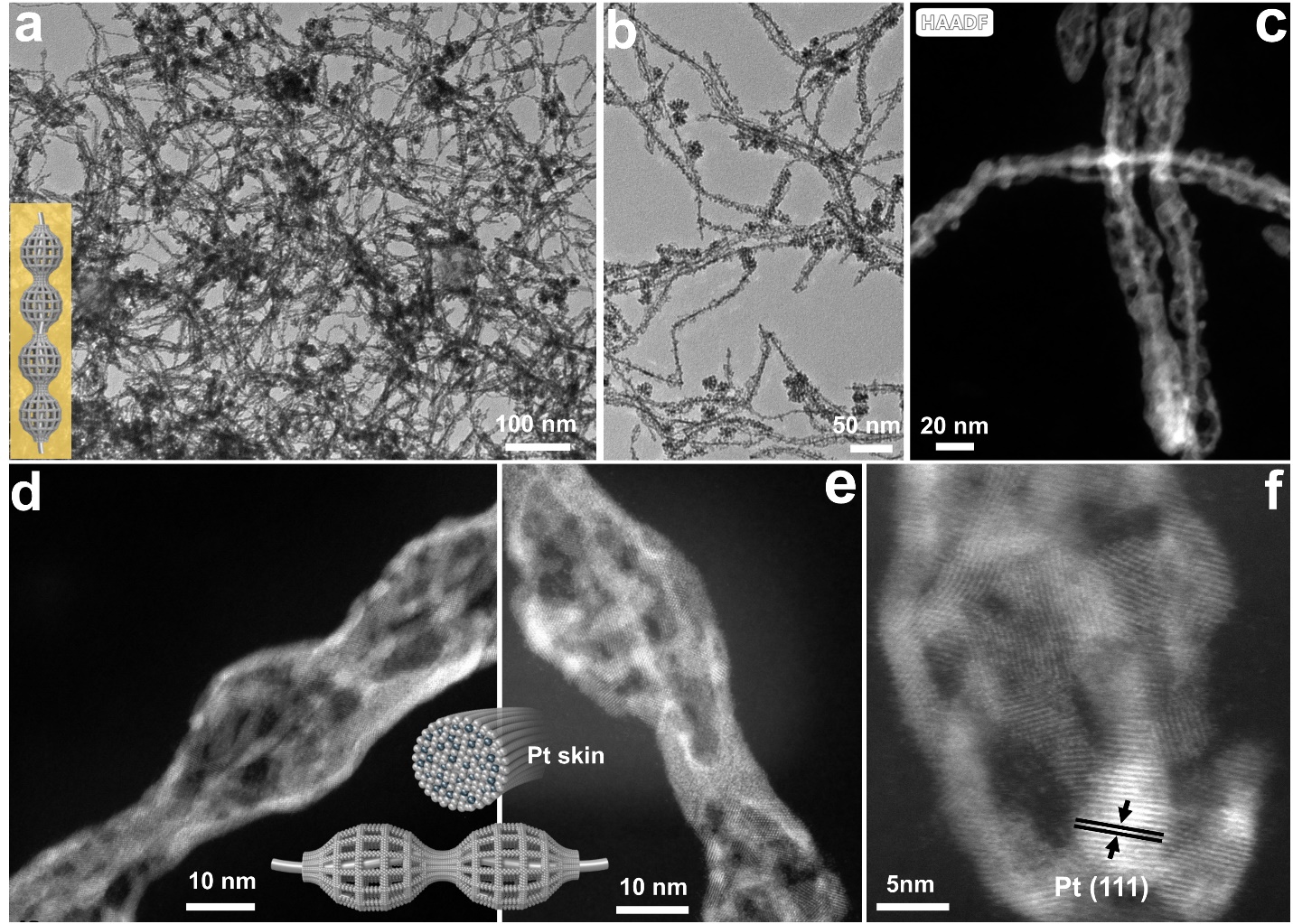
**Supplementary Figure 9.** Representative (**a**, **b**) TEM images, (**c**-**f**) HAADF-STEM images of Pt@Pt-skin Pt_3_Ni CSFWs.


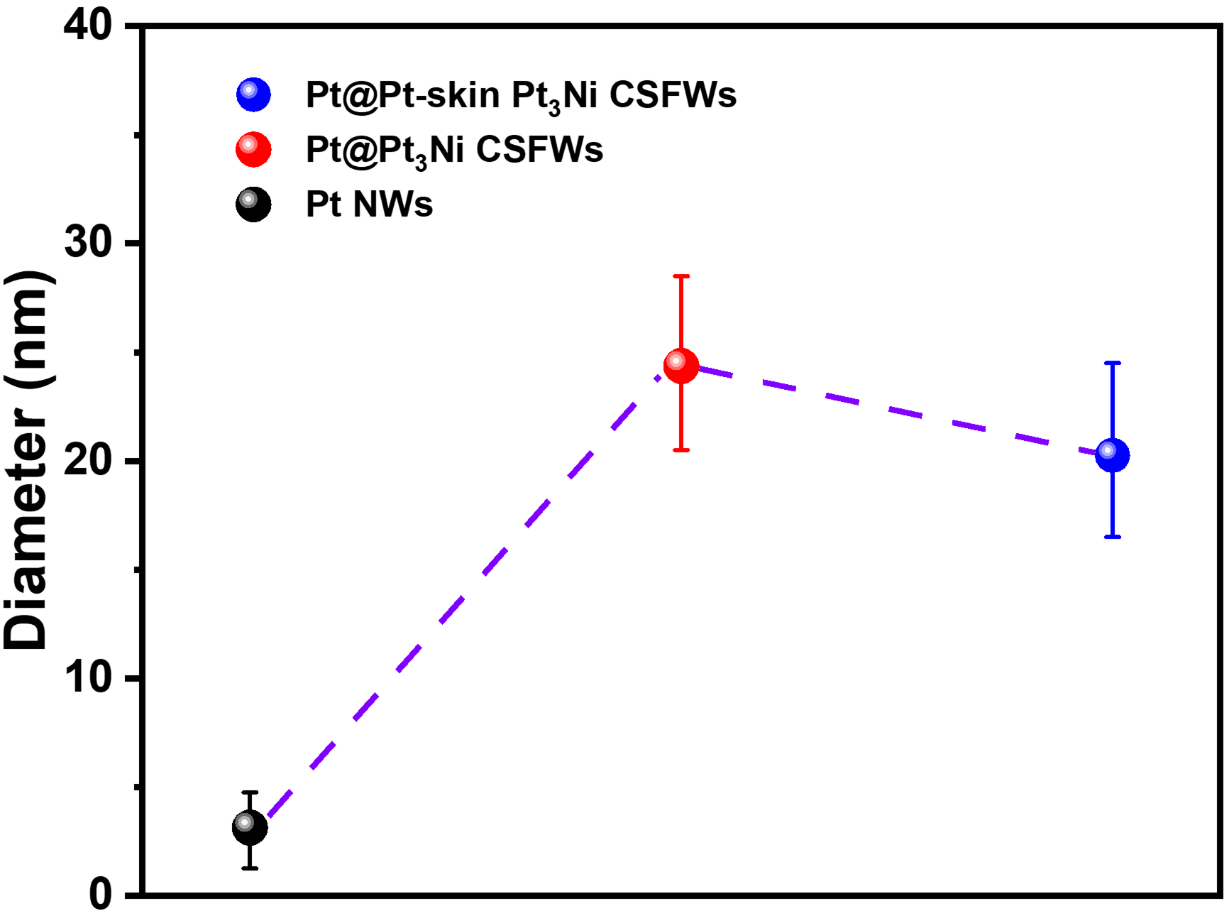
**Supplementary Figure 10.** The size distribution of initially formed Pt nanowires, Pt@Pt-Ni alloy CSNWs and final mesoporous Pt@Pt-skin Pt_3_Ni CSFWs. The average diameter of initially formed Pt nanowires, Pt@Pt-Ni alloy CSNWs and final mesoporous Pt@Pt-skin Pt_3_Ni CSFWs is 3.3 $\pm$ 1 nm, 24.3 $\pm$ 3 nm, 20.3 $\pm$ 3 nm, respectively. The error bars in the figure are obtained from the TEM images of each sample.


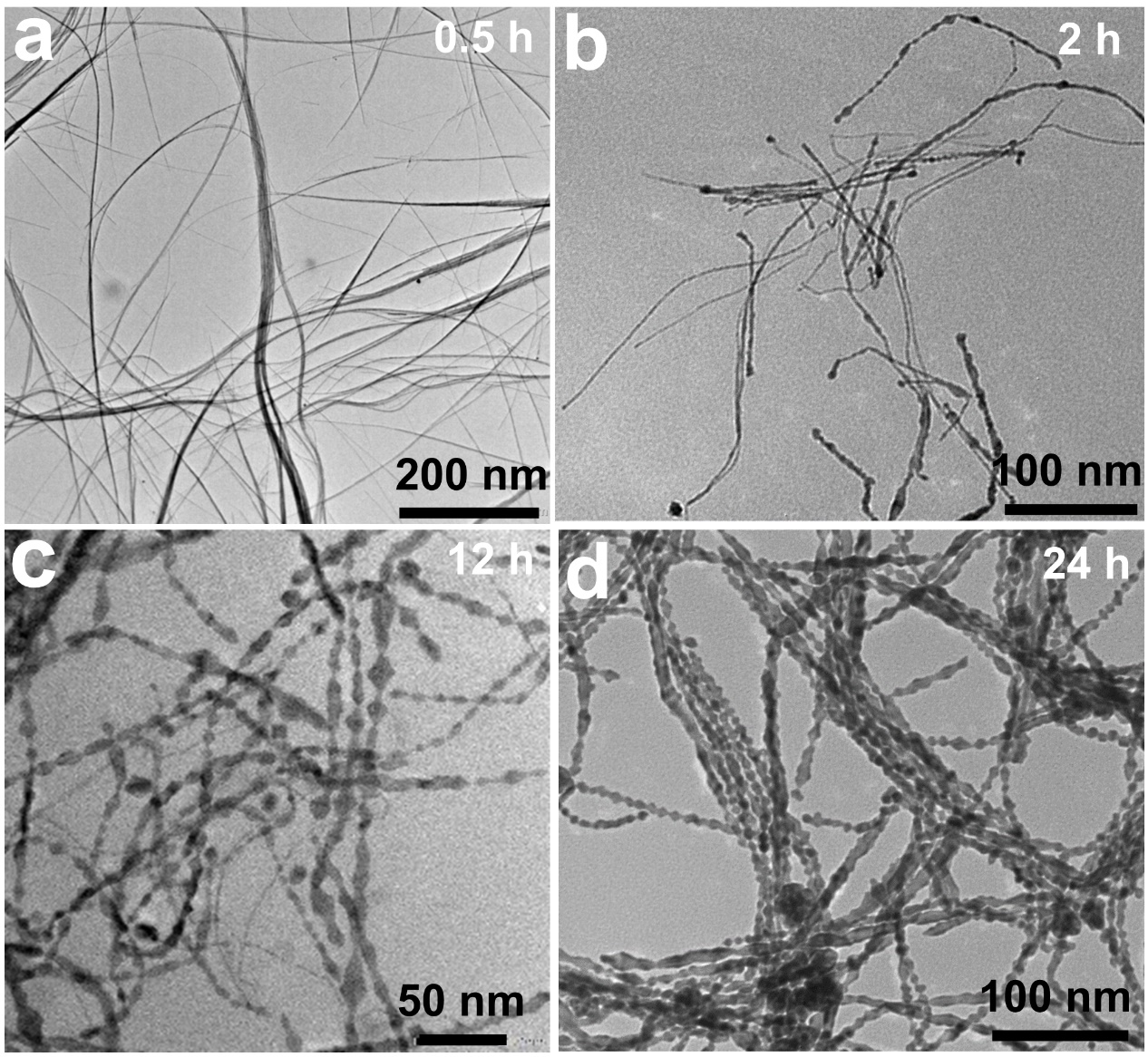
**Supplementary Figure 11**. TEM images of the samples with the same reaction conditions as those of Pt@Pt-Ni alloy CSNWs except the reaction time of **(a)** 0.5 h; **(b)** 2 h; **(c)** 12 h; **(d)** 24 h. the time-dependent morphology changes results reveal that the formation of the well-defined Pt@Pt-Ni CSNWs experienced the initial formation of ultrathin Pt nanowires (0.5 h), the deposition of Ni-rich phase onto the performed Pt nanowires (2 h-12h), and finally complete reduction of Pt/Ni precursors to form nanogourd-string-like Pt@Pt-Ni alloy CSNWs (24 h).


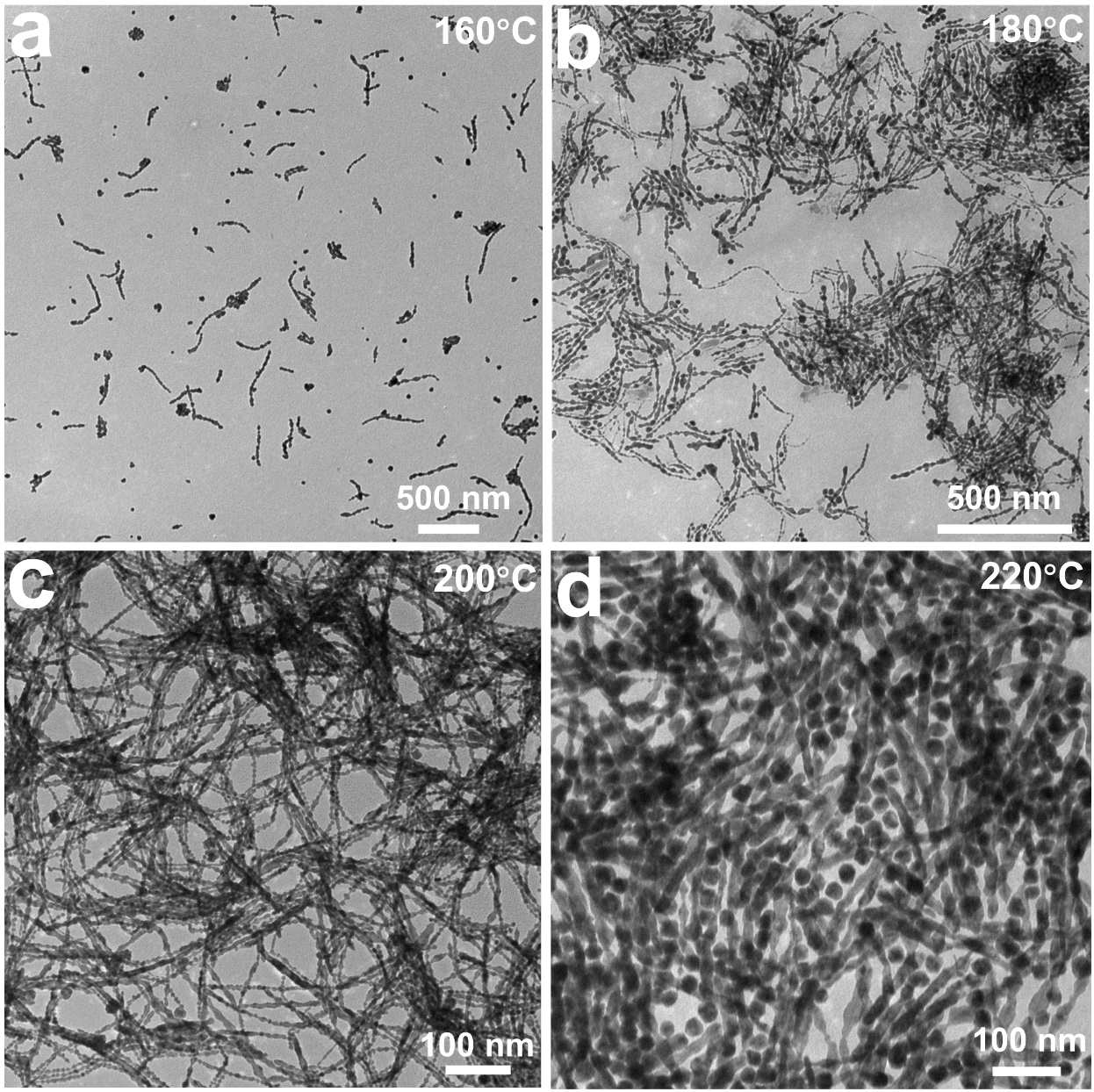
**Supplementary Figure 12**. TEM images of the samples with the same reaction conditions as those of Pt@Pt-Ni alloy CSNWs except the reaction temperature of **(a)** 160°C ; **(b)** 180°C ; **(c)** 200°C; **(d)** 220°C.


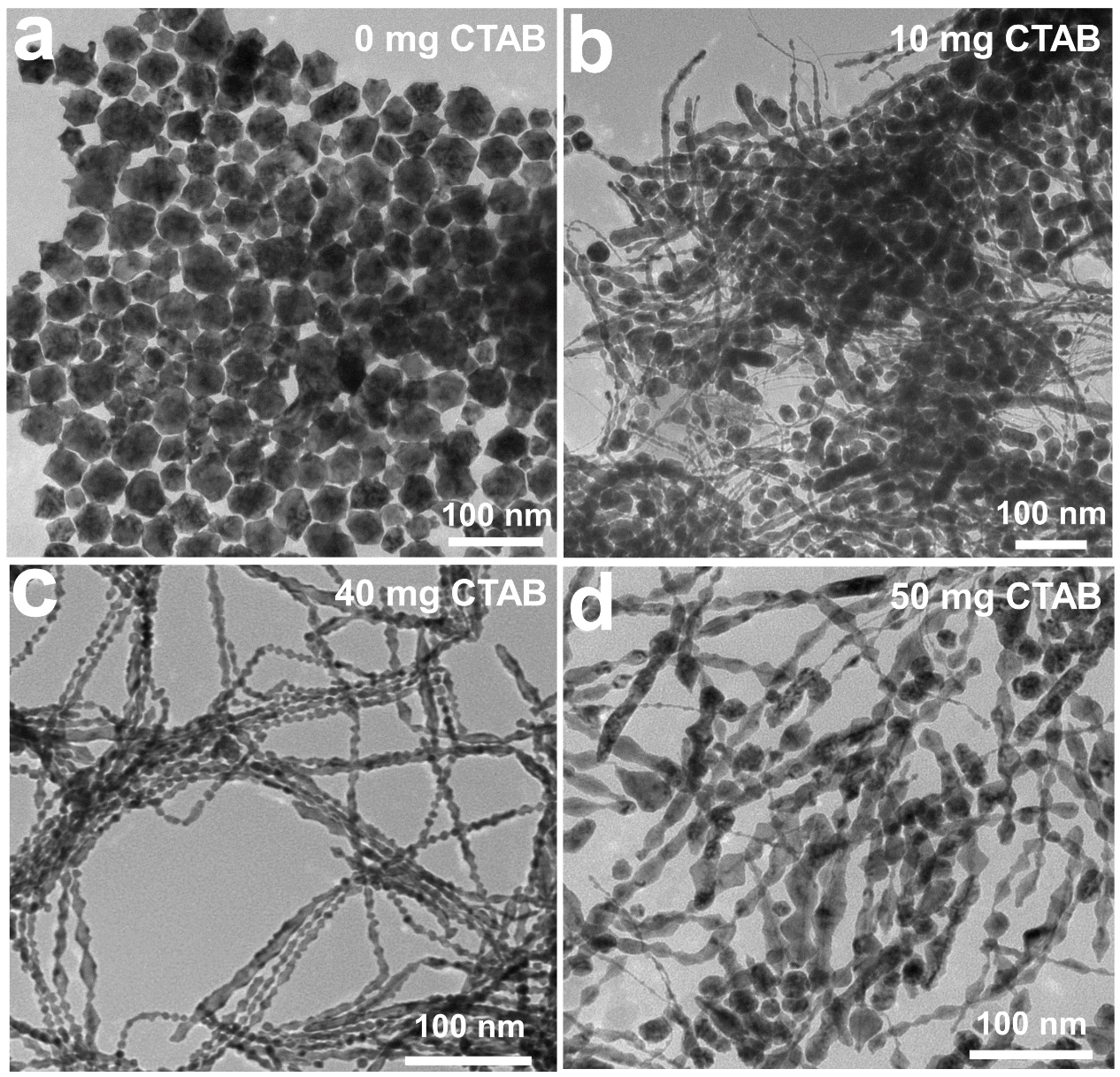
**Supplementary Figure 13**. TEM images of the samples with the same reaction conditions as those of Pt@Pt-Ni alloy CSNWs except the use of **(a)** 0 mg CTAB; **(b)** 10 mg CTAB; **(c)** 40 mg CTAB; **(d)** 50 mg CTAB.


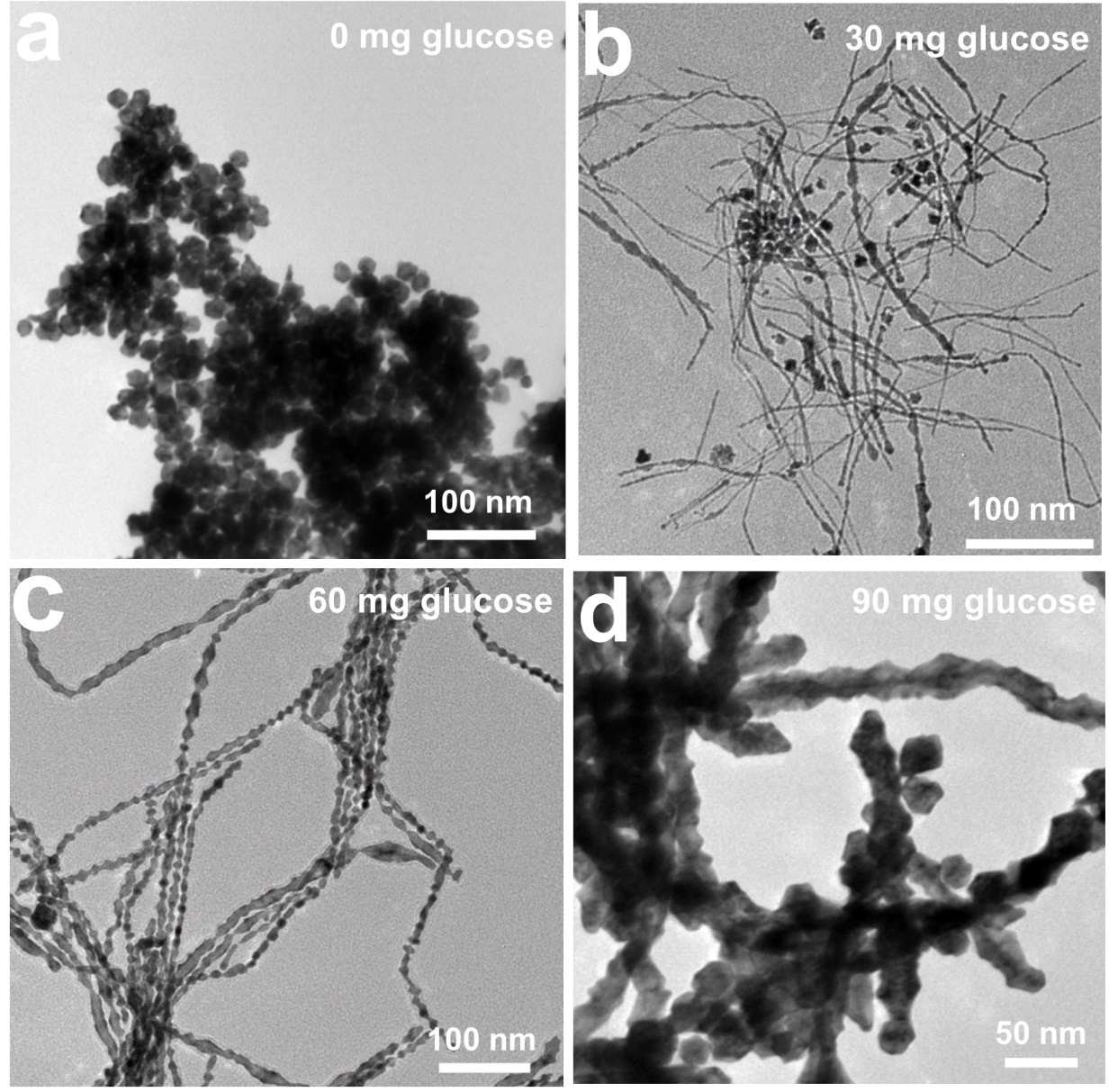
**Supplementary Figure 14**. TEM images of the samples with the same reaction conditions as those of Pt@Pt-Ni alloy CSNWs except the use of **(a)** 0 mg glucose; **(b)** 30 mg glucose; **(c)** 60 mg glucose; **(d)** 90 mg glucose.

Glucose as a reducing agent is critical to the final morphology of the catalyst. In the absence of glucose, irregular nanoparticles with agglomeration were obtained; When the amount of glucose increased to 30 mg, Pt-Ni nanowires with uneven morphology started to form; When the amount of glucose further increased to 60 mg, Pt@Pt-Ni alloy CSNWs with uniform morphology were yielded with diameters of around 24 nm. However, an excessive amount of glucose (90 mg) will result in irregular Pt@Pt-Ni alloy nanowires accompanied by serious agglomeration.


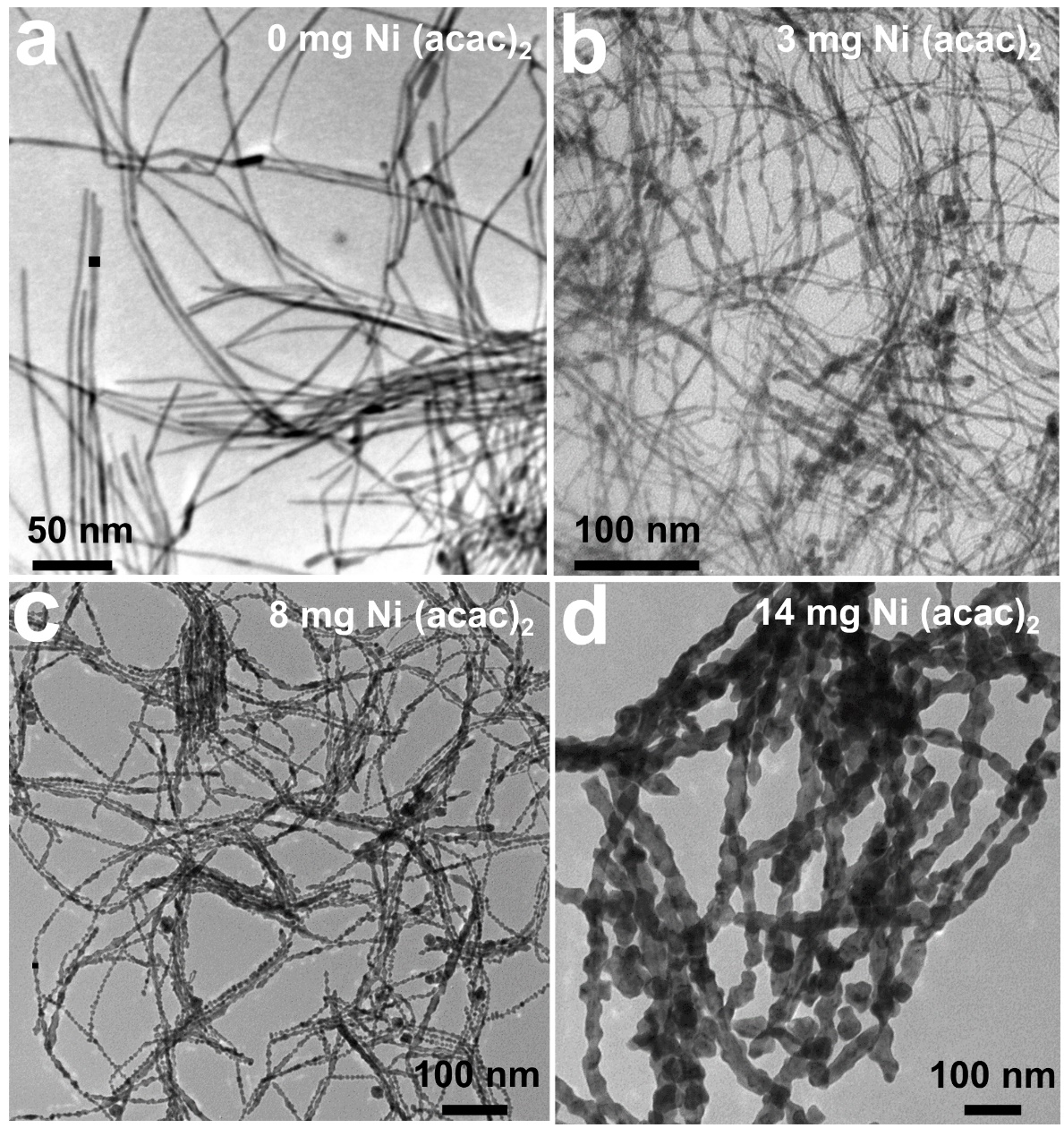
**Supplementary Figure 15**. TEM images of the samples with the same reaction conditions as those of Pt@Pt-Ni alloy CSNWs except the use of **(a)** 0 mg Ni (acac)_2_; **(b)** 3 mg Ni (acac)_2_; **(c)** 8 mg Ni (acac)_2_; **(d)** 14 mg Ni (acac)_2_.


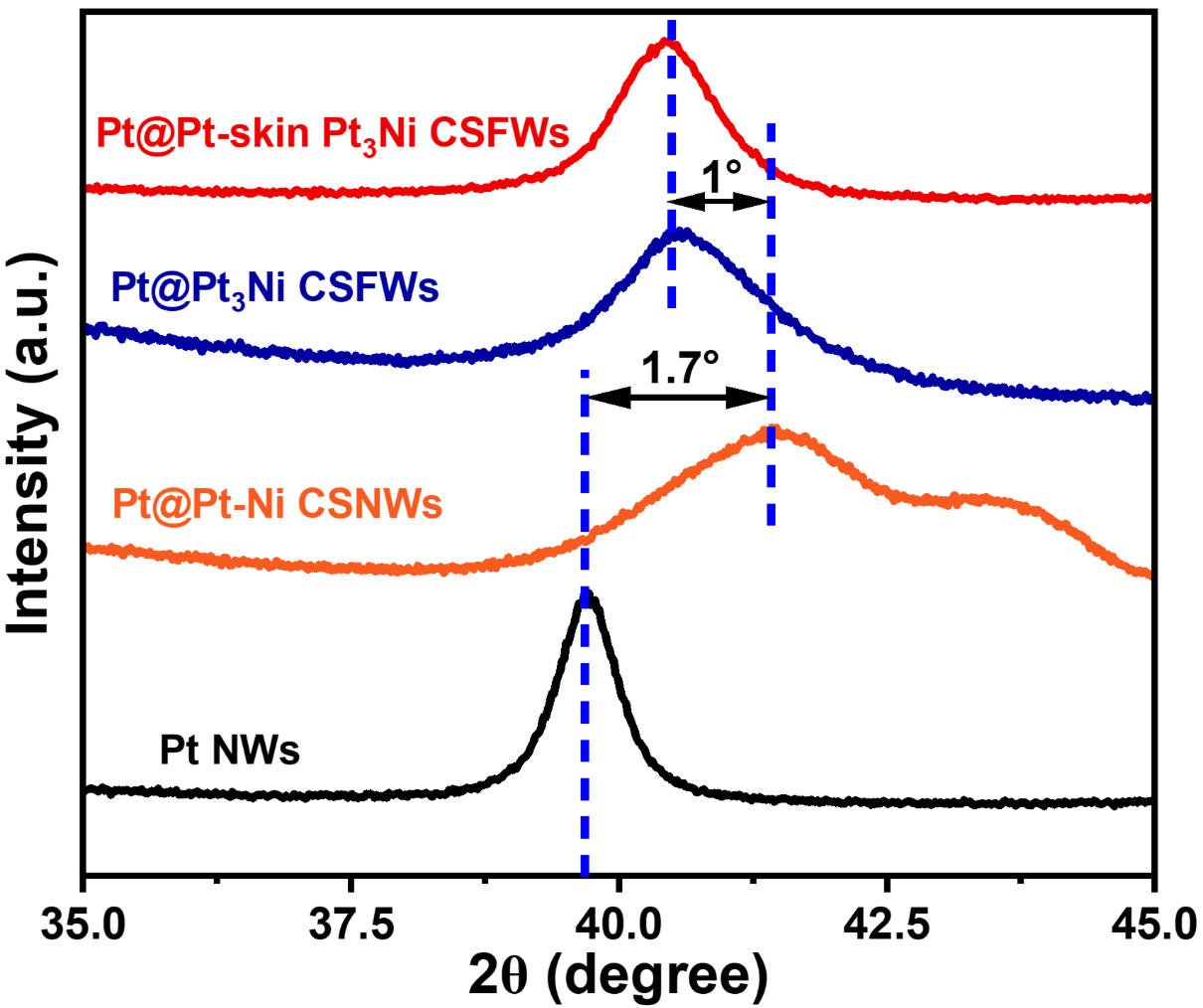
**Supplementary Figure 16.** Enlarged Ex-situ XRD patterns of the samples evolved from Pt nanowires into mesoporous Pt@Pt-skin Pt_3_Ni CSFWs.


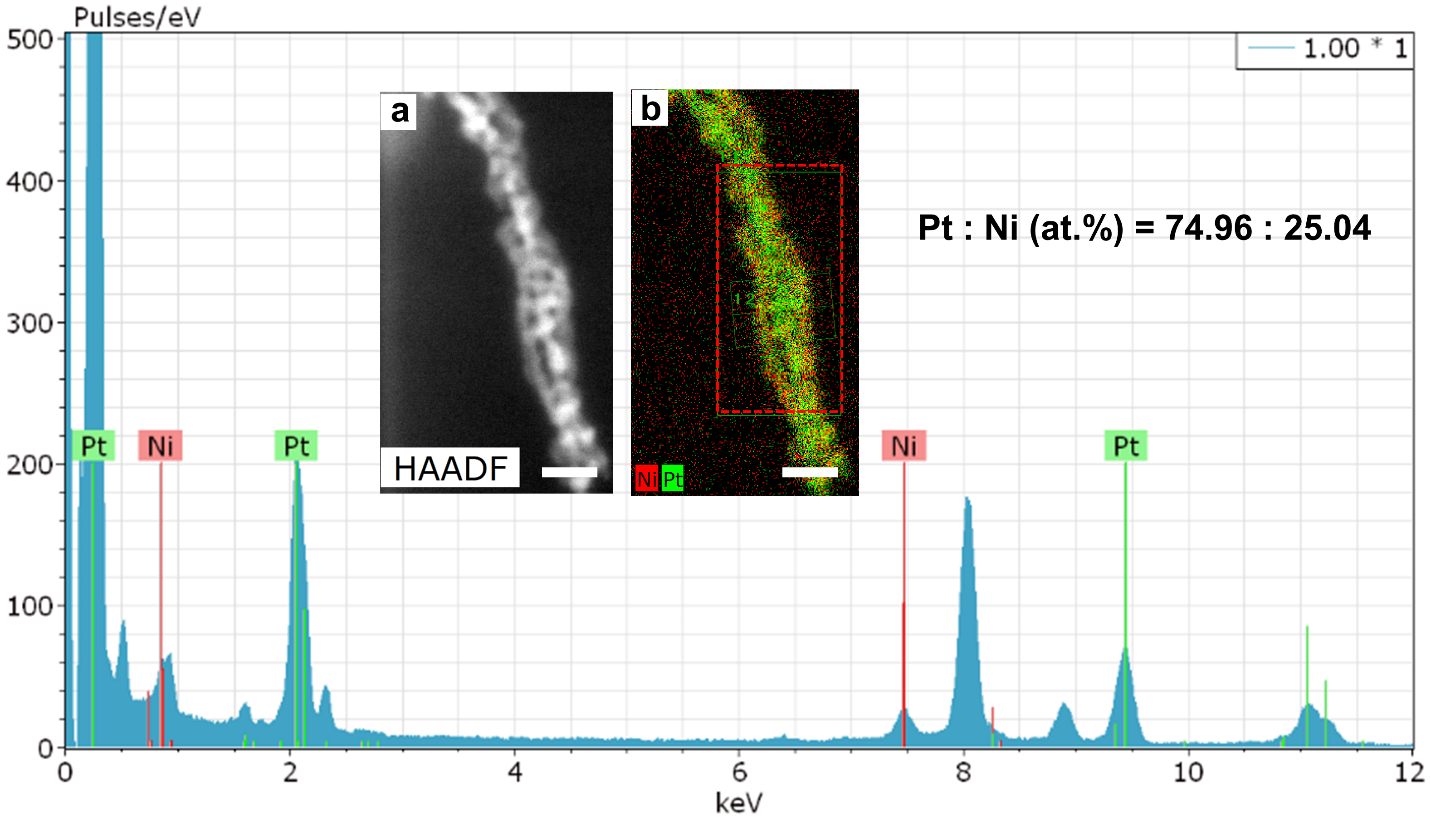
**Supplementary Figure 17.** STEM-EDS profile of Pt@Pt-skin Pt_3_Ni CSFWs from the area marked by the red dashed square, indicating that the overall Pt /Ni atomic ratio is approximately 3:1, scale bars in (a) and (b), 20 nm.


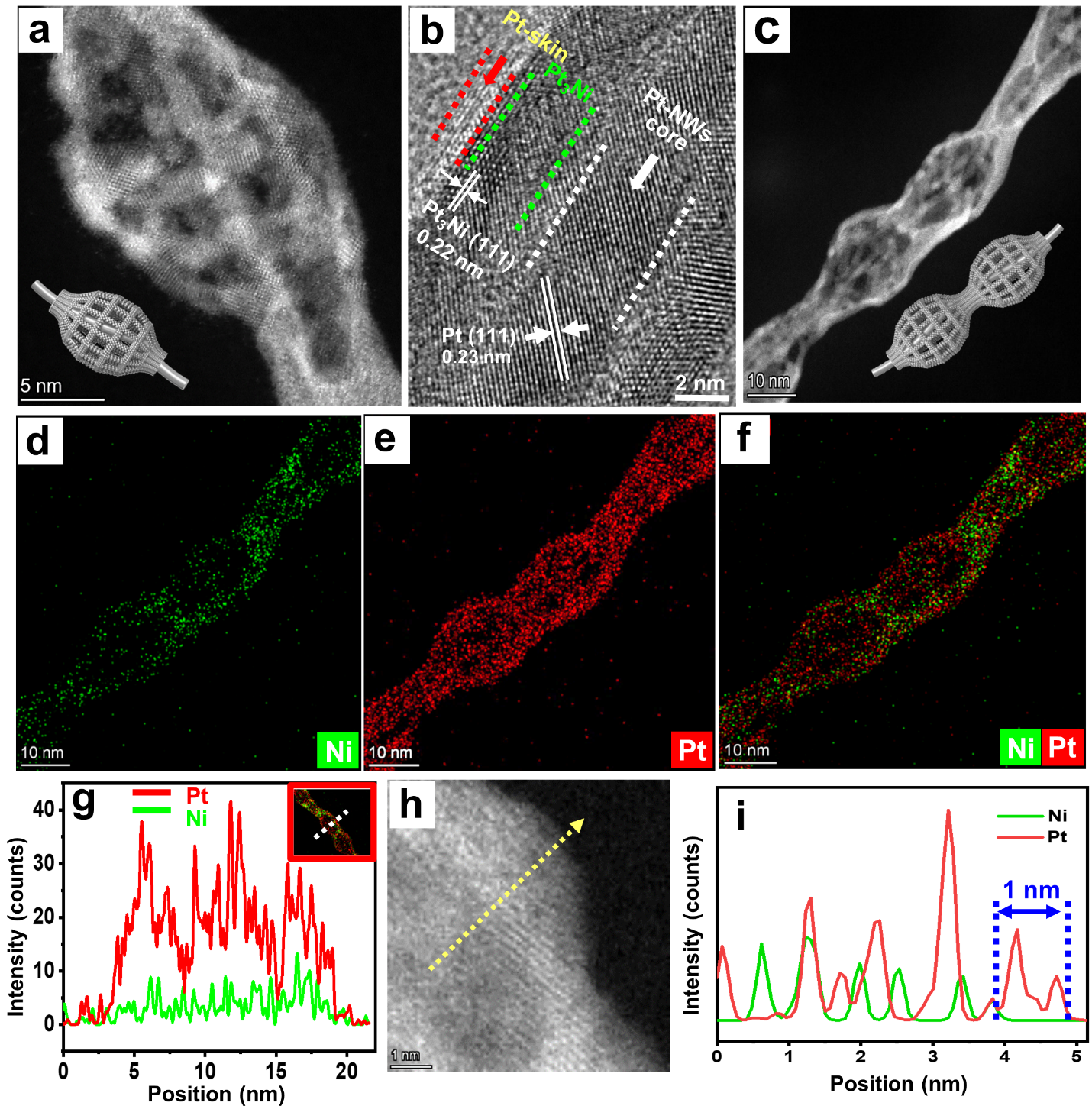
**Supplementary Figure 18. (a-c)** HAADF-STEM and HRTEM images; **(d-f)** STEM-EDX elemental mapping; **(g)** STEM-EDX line-scanning profile of an individual mesoporous Pt@Pt-skin Pt_3_Ni CSFWs; **(h)** Local magnification HRTEM images of an individual mesoporous Pt@Pt-skin Pt_3_Ni CSFWs; **(i)** STEM-EDX line-scanning profile shown by the yellow line in figure h.


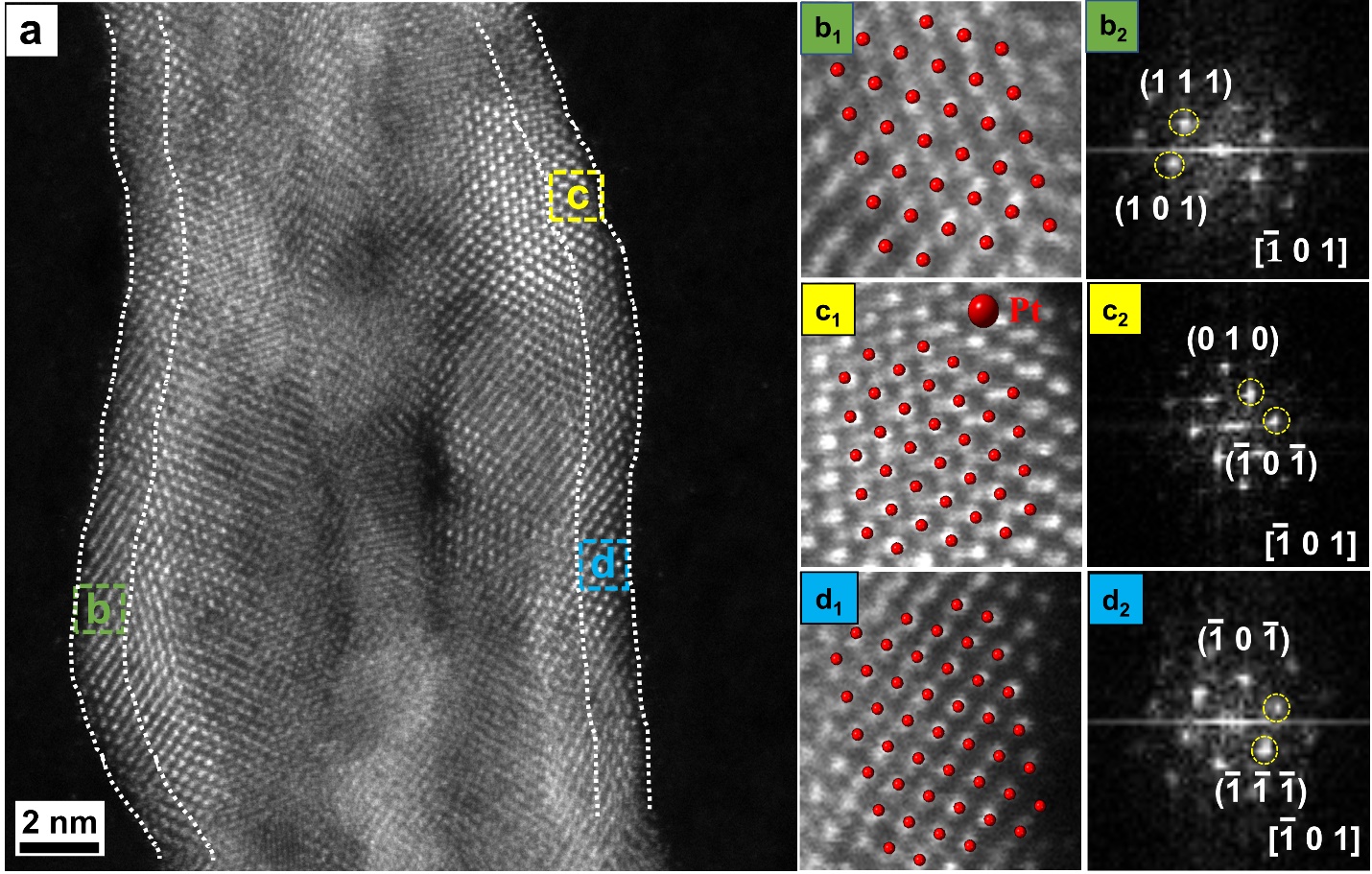
**Supplementary Figure 19. (a)** HAADF-STEM image of an individual mesoporous Pt@Pt-skin Pt_3_Ni CSFWs. **(b_1_-d_1_)** The corresponding enlarged view of the rectangular marked area in **(a). (b_2_-d_2_)** The corresponding FFT pattern of **(b_1_), (c_1_)** and **(g_1_)**, respectively.


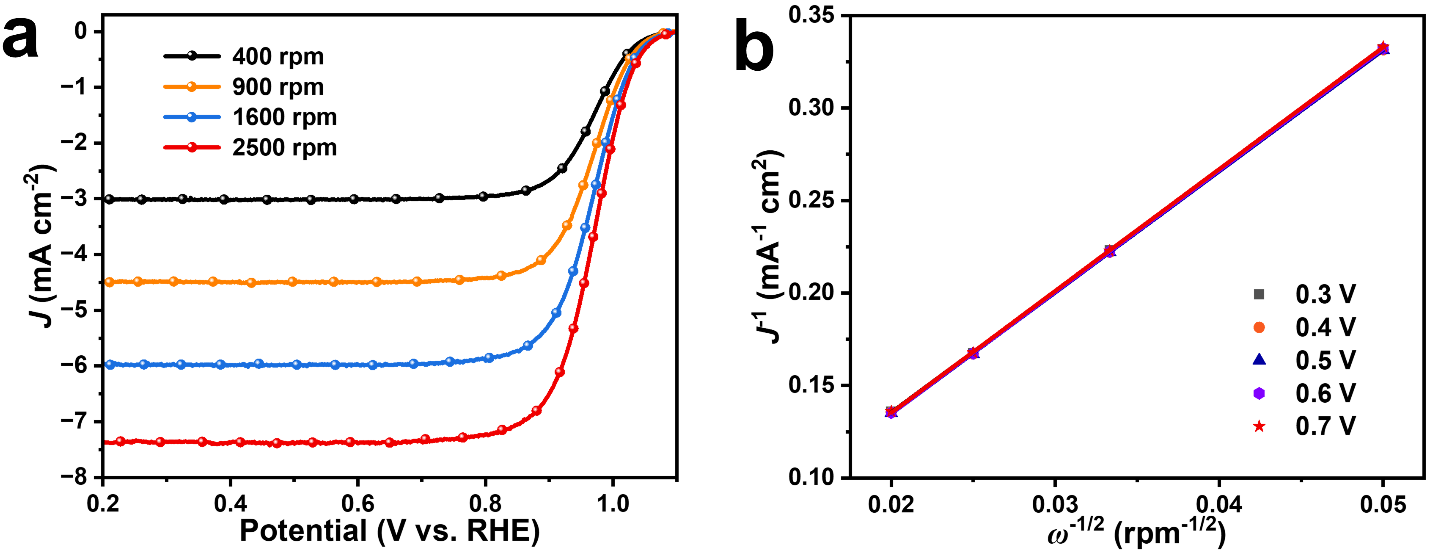
**Supplementary Figure 20.** (**a**) ORR polarization curves of mesoporous Pt@Pt-skin Pt_3_Ni CSFWs/C at different rotating rates. For the mesoporous Pt@Pt-skin Pt_3_Ni CSFWs, the Pt loading on RDE is 6.5 µg cm^–2^. (**b**) The resulting Koutecky-Levich (K-L) plots from Supplementary Figure 20a. The number of transfer electrons calculated from K-L equation is approximately 4.07 for Pt@Pt-skin Pt_3_Ni CSFWs, which suggests an efficient 4-electrons reaction process on the Pt@Pt-skin Pt_3_Ni CSFWs catalyst.


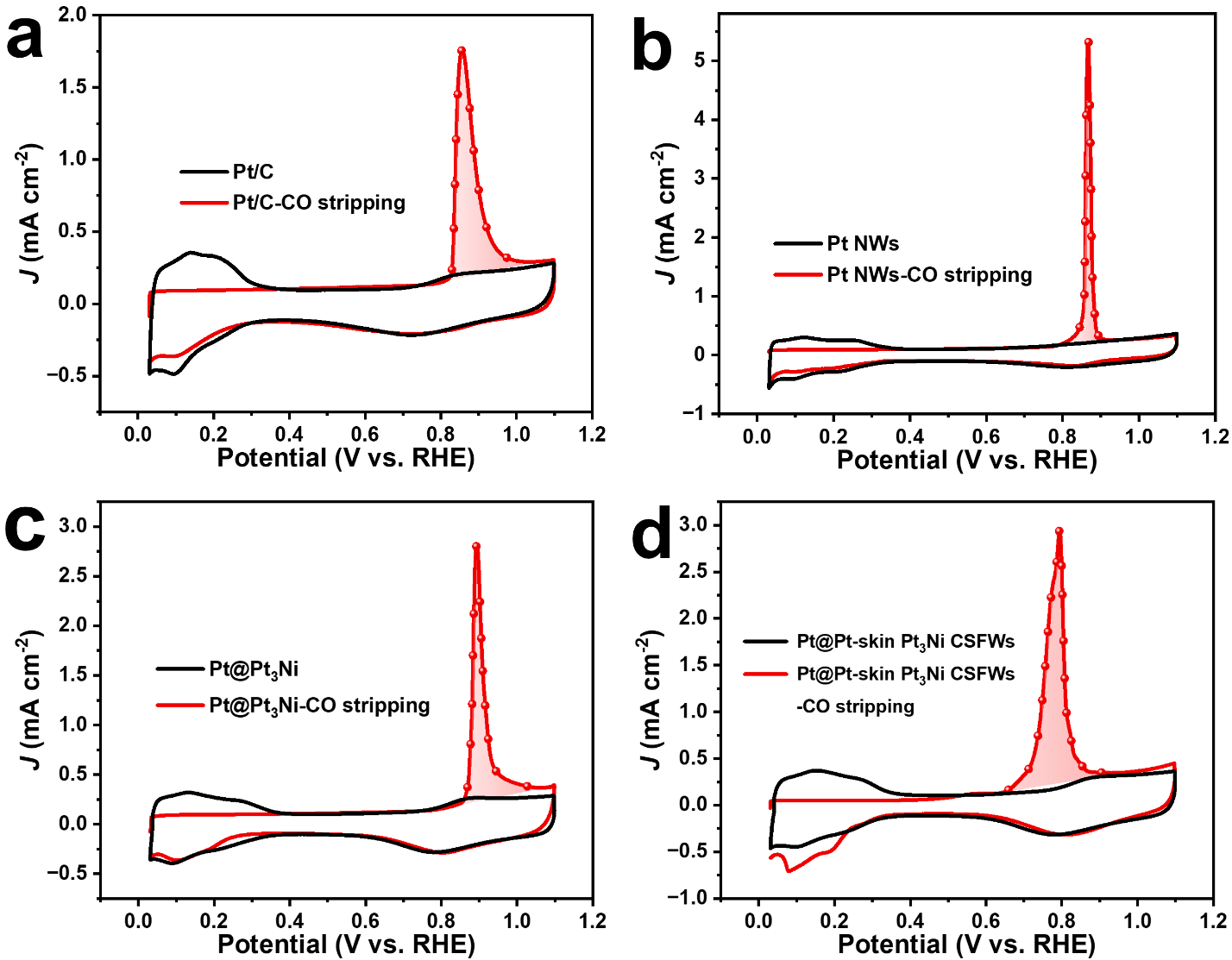
**Supplementary Figure 21.** CV and CO stripping curves of **(a)** Pt/C, **(b)** Pt NWs/C, **(c)** mesoporous Pt@Pt_3_Ni CSFWs/C and **(d)** mesoporous Pt@Pt-skin Pt_3_Ni CSFWs/C, respectively. The Pt loading for all catalysts on RDE is 6.5 µg cm^–2^.


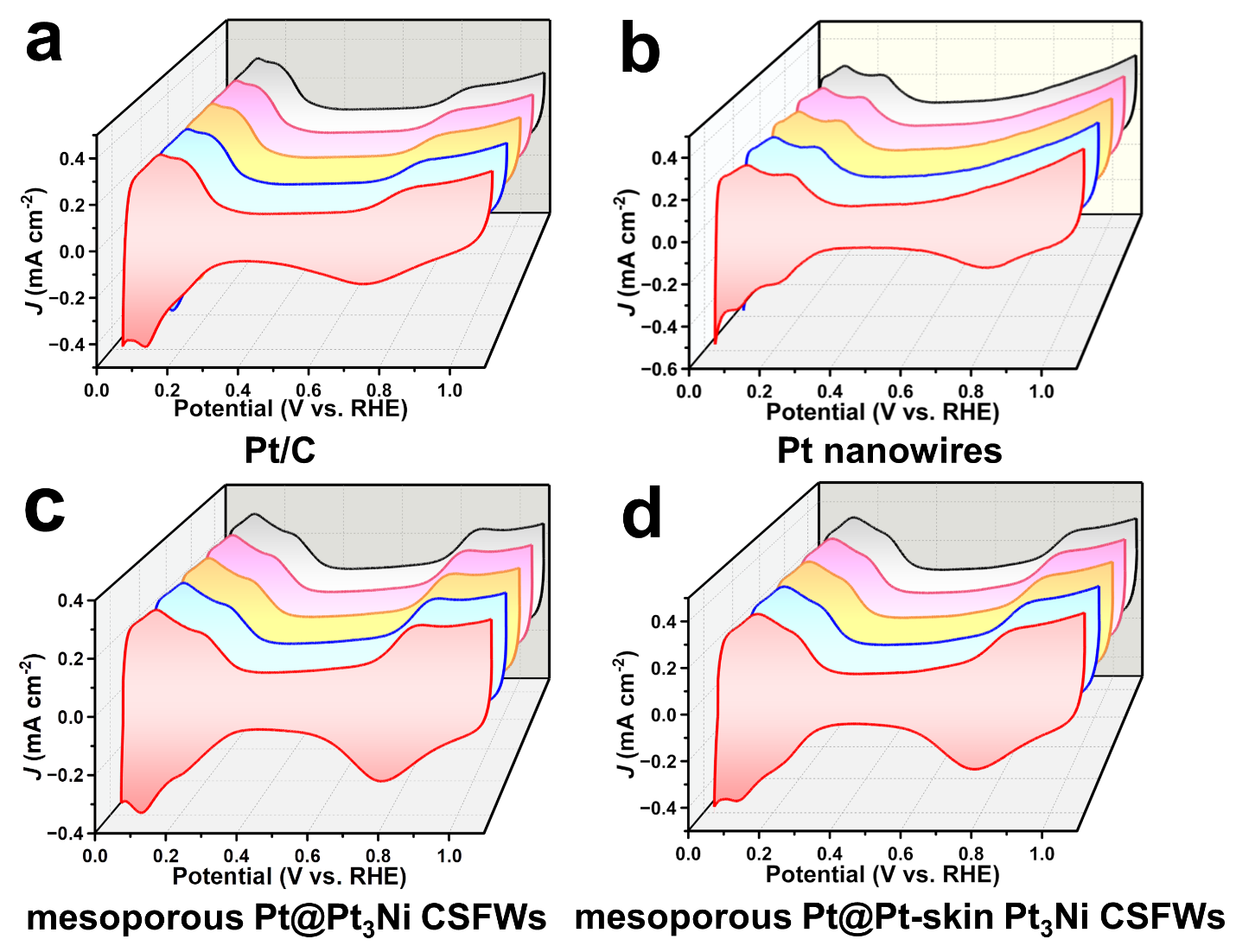
**Supplementary Figure 22.** CV curves of **(a)** Pt/C, **(b)** Pt NWs, **(c)** mesoporous Pt@Pt_3_Ni CSFWs and **(d)** mesoporous Pt@Pt-skin Pt_3_Ni CSFWs based on 5 independent thin-film electrodes in 0.1 M HClO_4_ electrolyte. The Pt loading maintains 6.5 µg cm^-2^ for all catalysts on RDE.


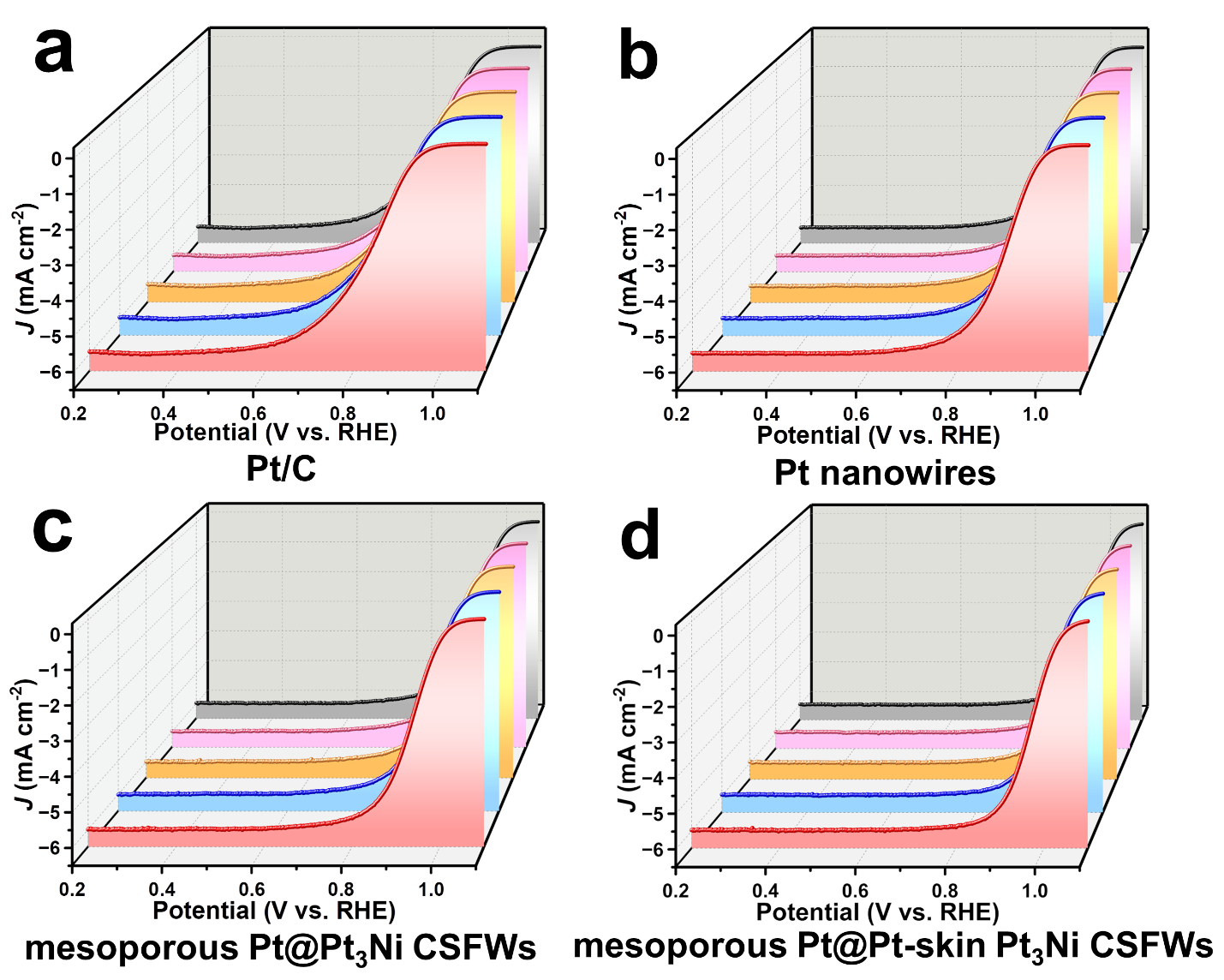
**Supplementary Figure 23.** ORR polarization curves of **(a)** Pt/C, **(b)** Pt NWs, **(c)** mesoporous Pt@Pt_3_Ni CSFWs and **(d)** mesoporous Pt@Pt-skin Pt_3_Ni CSFWs based on 5 independent thin-film electrodes in 0.1 M HClO_4_ electrolyte. The Pt loading for all catalysts on RDE is kept at 6.5 µg cm^-2^.


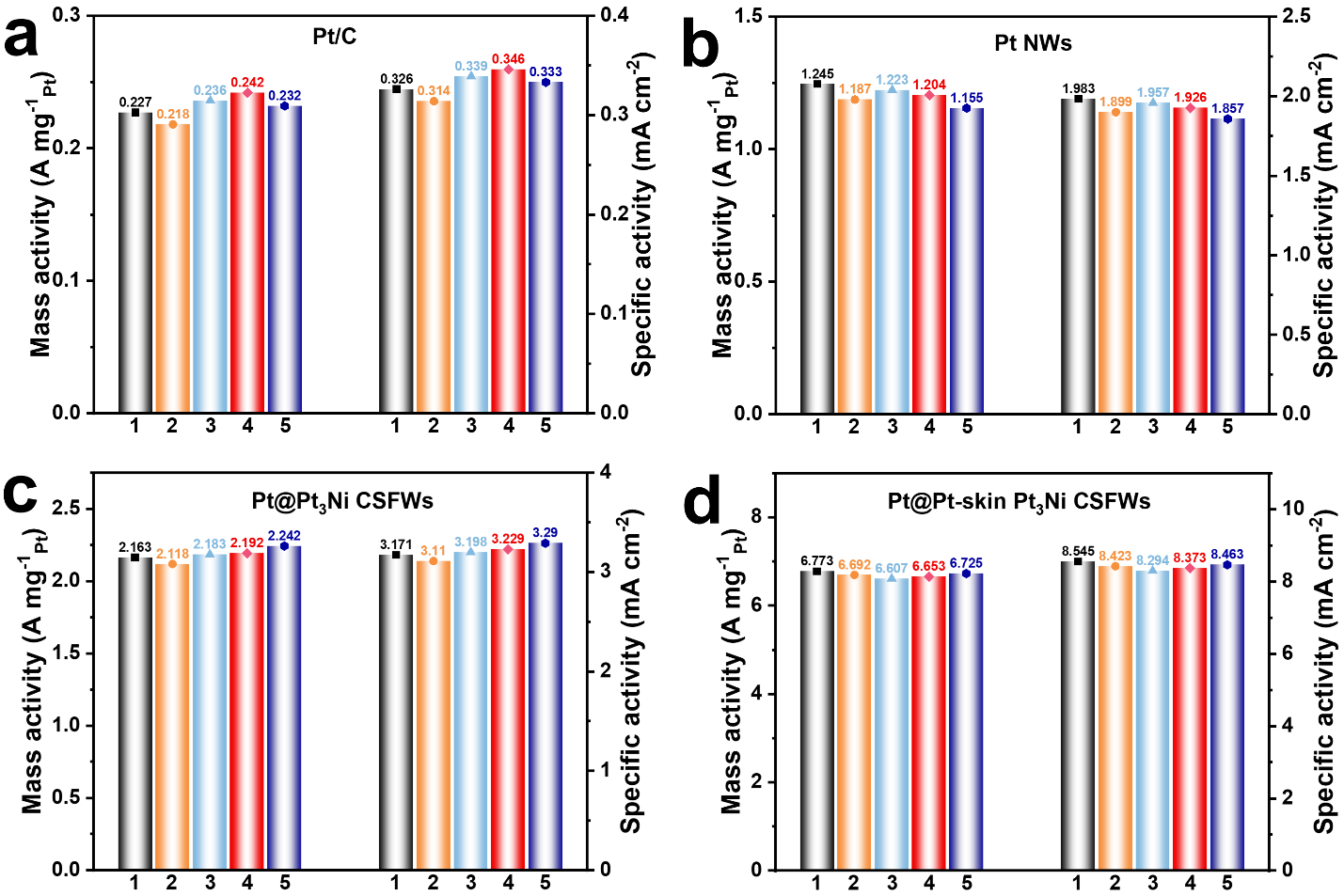
**Supplementary Figure 24.** Mass activity and specific activity of **(a)** Pt/C, **(b)** Pt NWs, **(c)** mesoporous Pt@Pt_3_Ni CSFWs and **(d)** mesoporous Pt@Pt-skin Pt_3_Ni CSFWs based on 5 independent thin-film electrodes in 0.1 M HClO_4_ electrolyte.


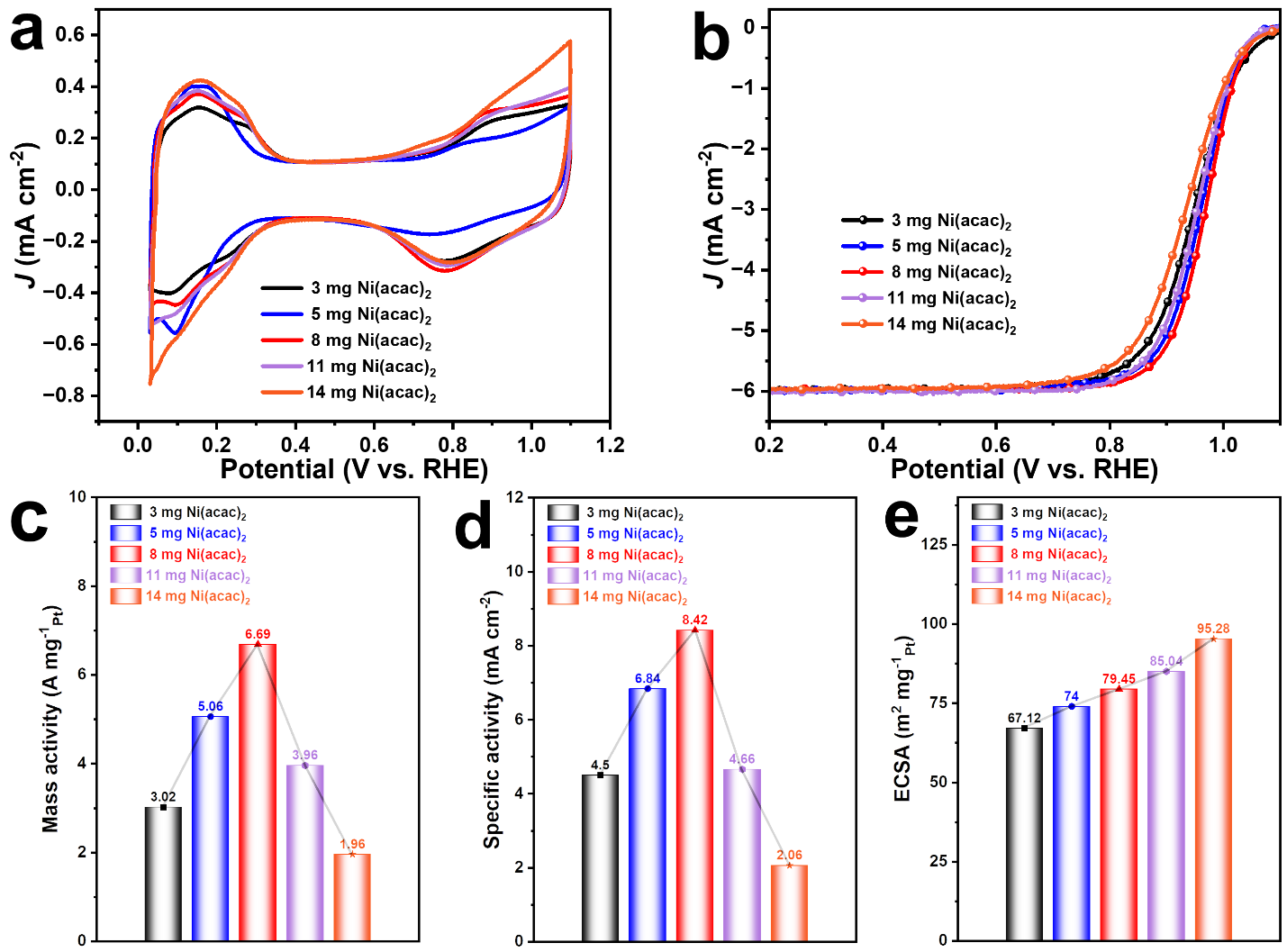
**Supplementary Figure 25.** Electrocatalytic performance of different mesoporous Pt@Pt-Ni CSFWs samples obtained from different Ni/Pt ratios**. (a)** CVs, **(b)** LSVs, **(c)** Mass activities, **(d)** Specific activities and **(e)** ECSA. All catalyst samples experienced the same acid treatment and heat treatment before the ORR test. The ORR results showed the volcano-shaped activity relationship, and the uniform mesoporous Pt@Pt-skin Pt_3_Ni CSFWs/C obtained by adding 8 mg Ni (acac)_2_ achieves the best ORR performance. For all catalysts, the Pt loading on RDE is 6.5 µg cm^-2^.


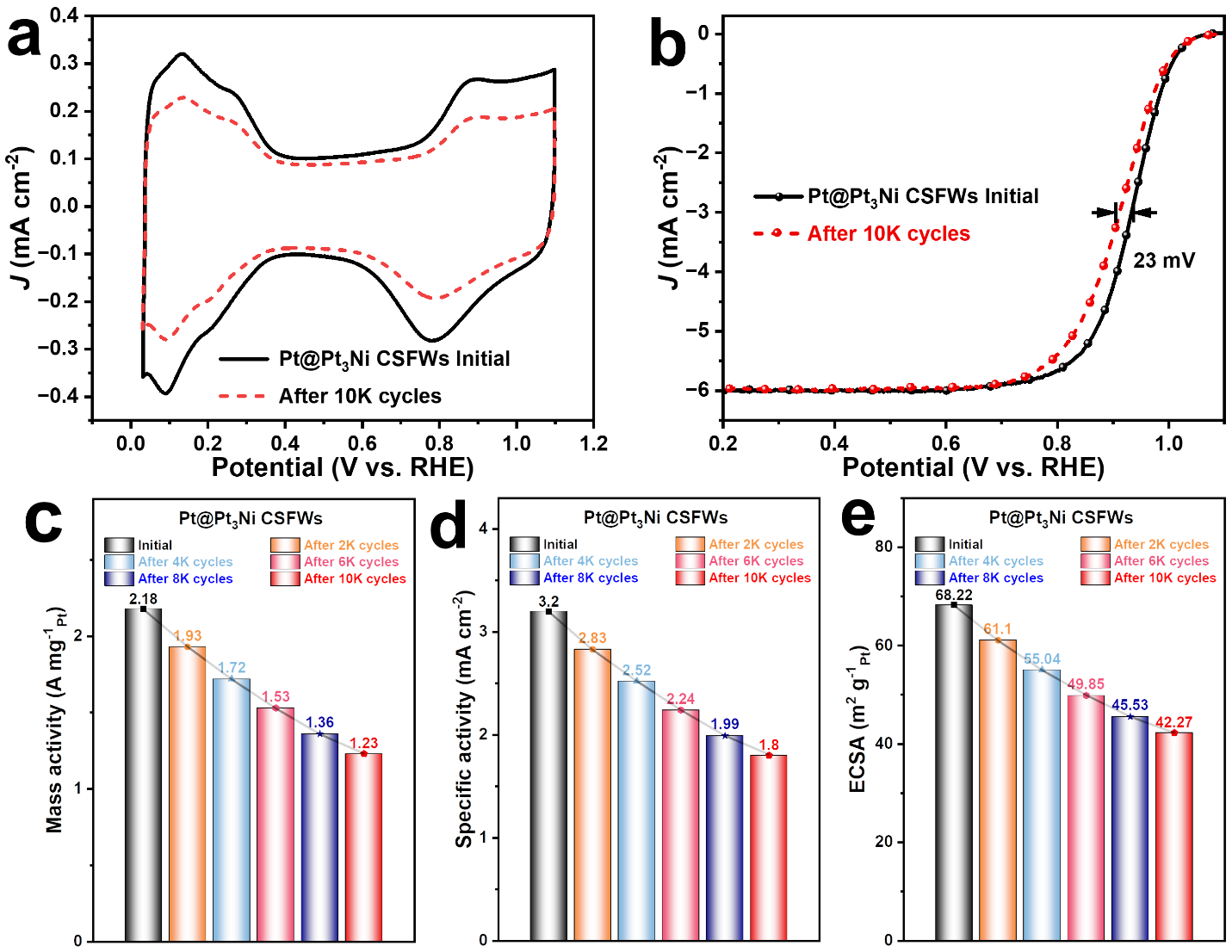
**Supplementary Figure 26.** The durability testing results for Pt@Pt_3_Ni CSFWs/C. **(a)** CV evolution before and after 10,000 potential cycles. **(b)** ORR polarization curve evolution before and after 10,000 potential cycles. For the Pt@Pt_3_Ni CSFWs/C, the Pt loading on RDE is 6.5 µg cm^-2^. **(c, d, e)** Mass activity, specific activity and ECSA evolutions, respectively, before and after different potential cycles.


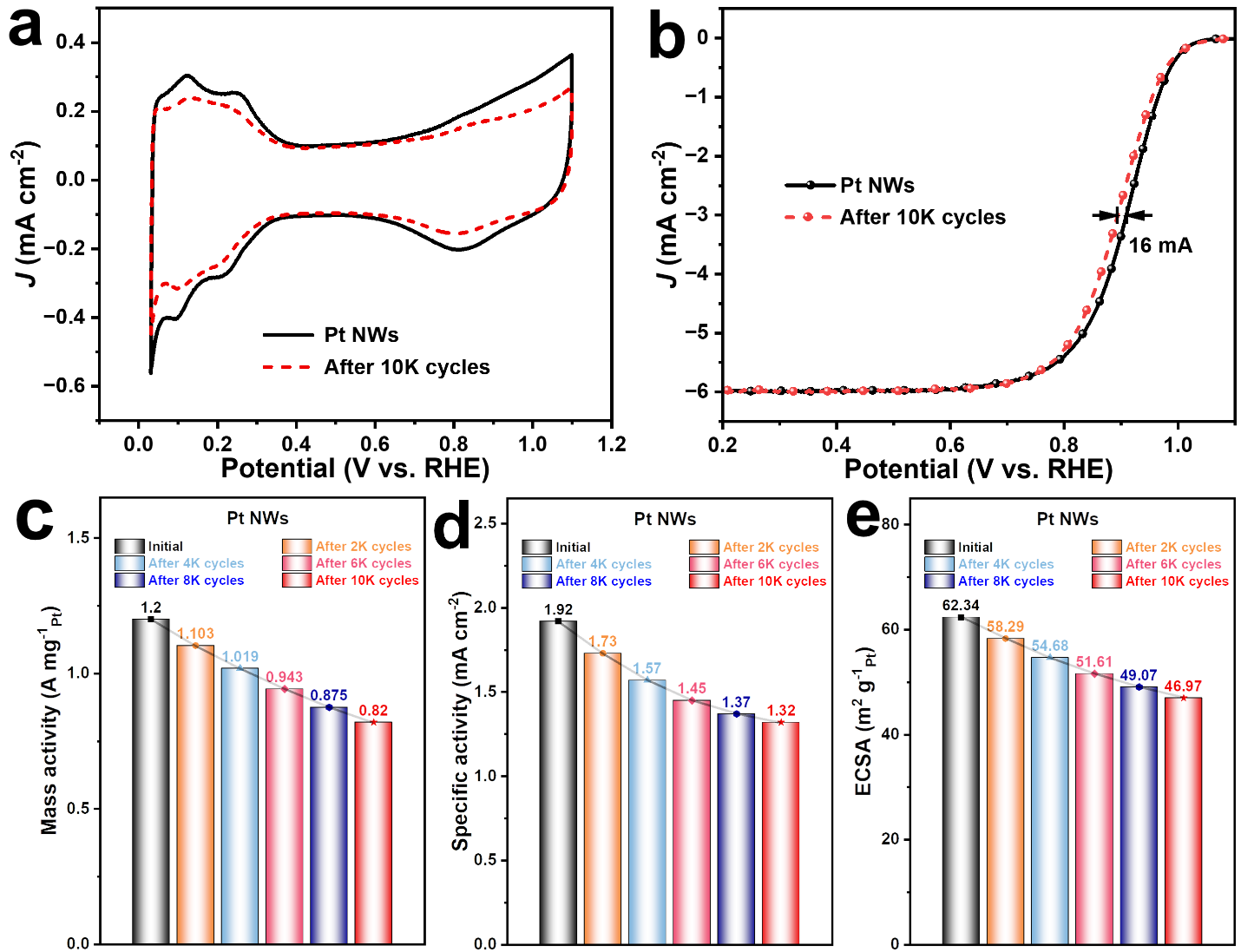
**Supplementary Figure 27.** The durability testing results for the Pt nanowires. **(a)** CV evolution before and after 10,000 potential cycles. **(b)** ORR polarization curve evolution before and after 10,000 potential cycles. For the Pt NWs/C, the Pt loading on RDE is 6.5 µg cm^-2^. **(c, d, e)** Mass activity, specific activity and ECSA evolutions, respectively, before and after different potential cycles.


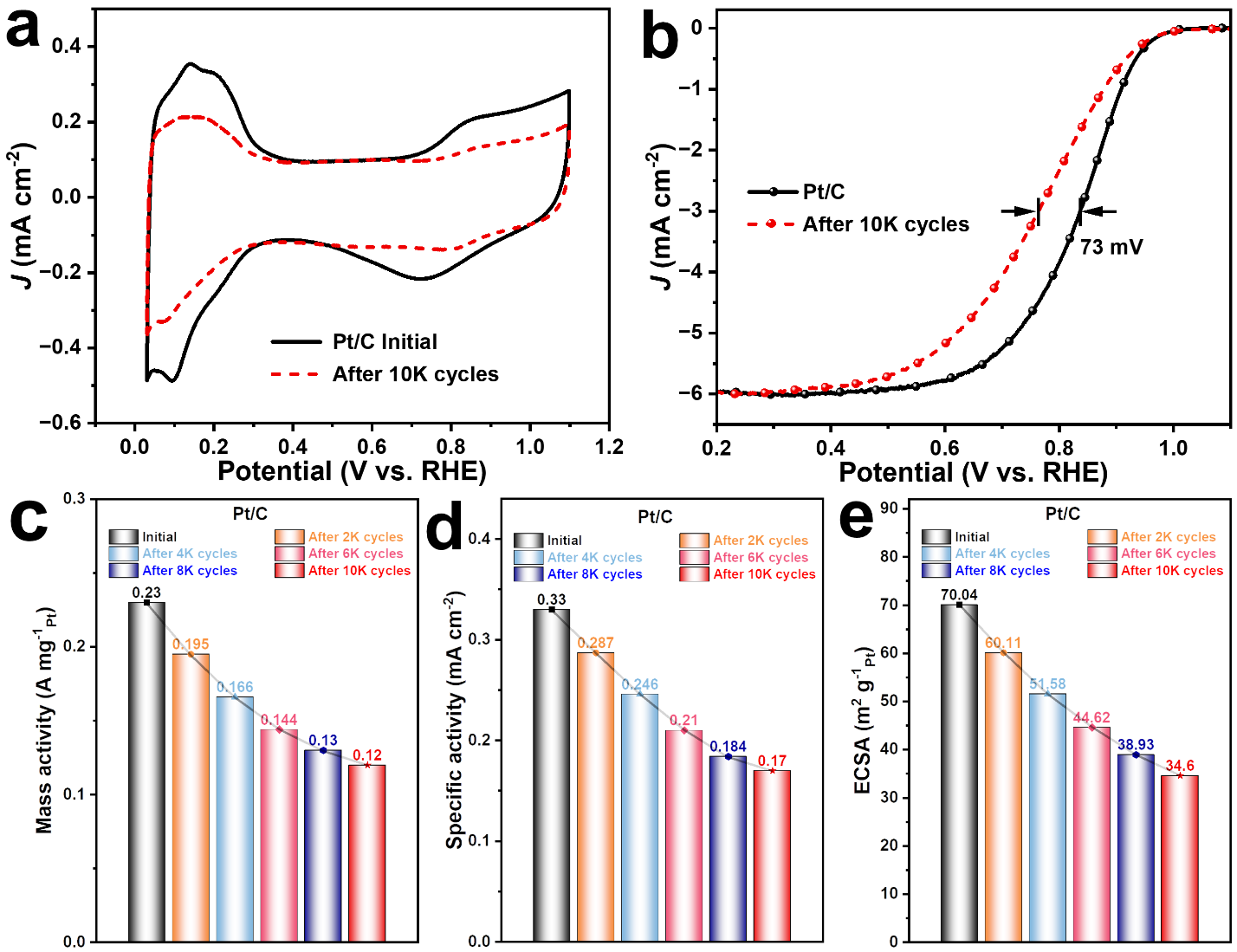
**Supplementary Figure 28.** The durability testing results for the Pt/C. **(a)** CV evolution before and after 10,000 potential cycles. **(b)** ORR polarization curve evolution before and after 10,000 potential cycles. For the Pt/C, the Pt loading on RDE is 6.5 µg cm^-2^. **(c, d, e)** Mass activity, specific activity and ECSA evolutions, respectively, before and after different potential cycles.


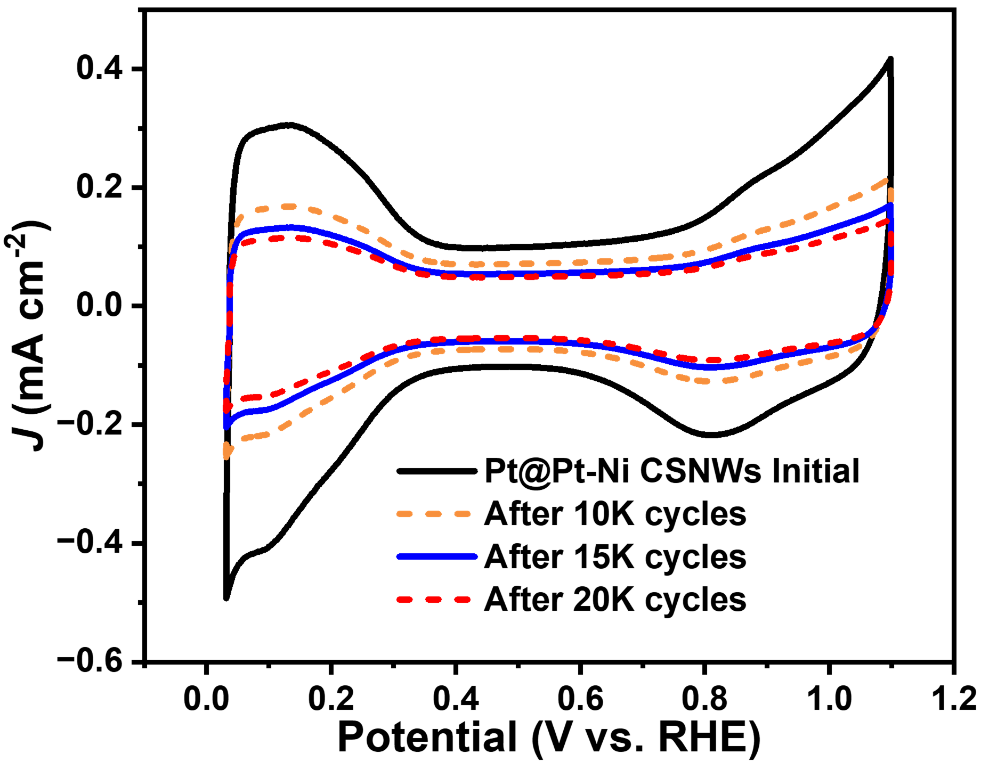
**Supplementary Figure 29.** CV evolutions for Pt@Pt-Ni alloy CSNWs/C before and after different potential cycles. For the Pt@Pt-Ni alloy CSNWs/C, the Pt loading on RDE is 6.5 µg cm^-2^.


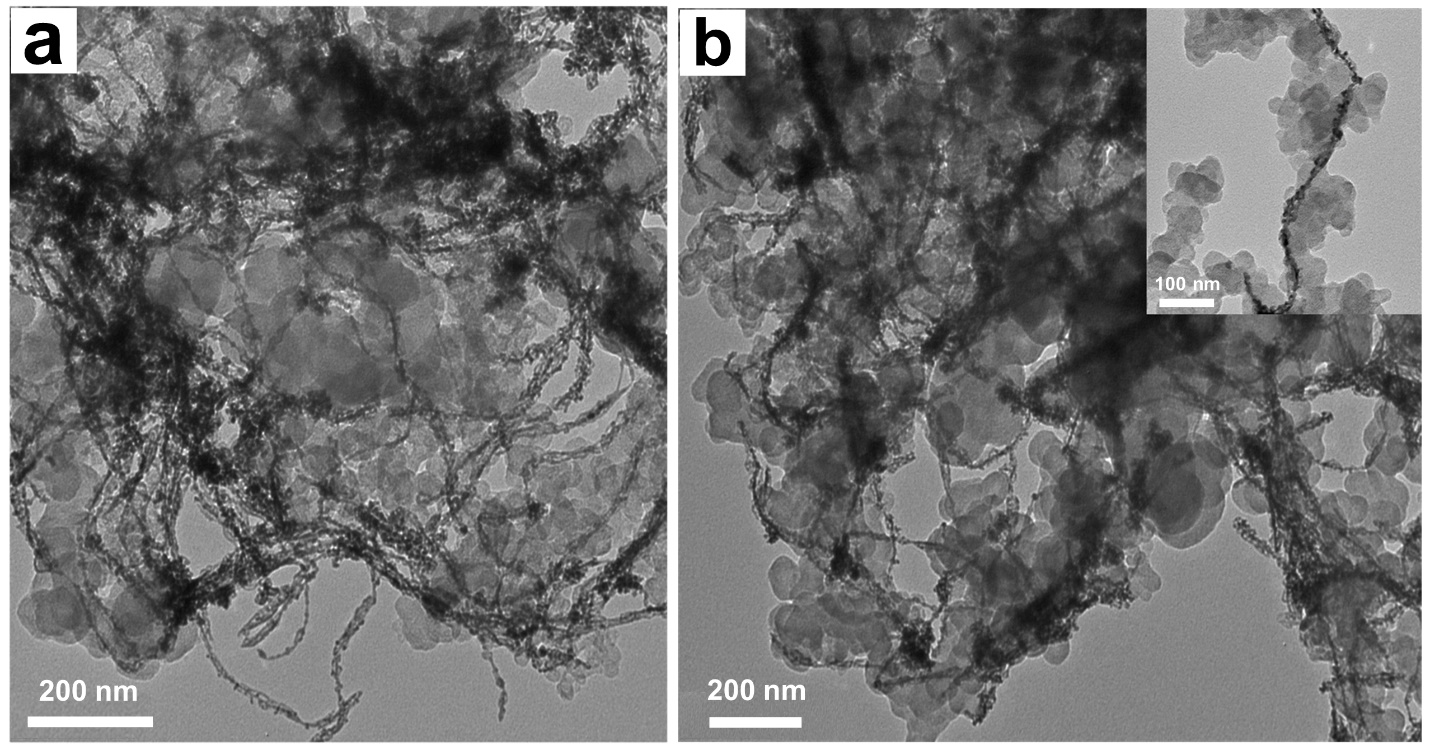
**Supplementary Figure 30.** TEM images of Pt@Pt-skin Pt_3_Ni CSFWs/C (**a**) before and (**b**) after 50,000 cycles’ durability test. TEM images showed negligible change in overall morphology or size of the Pt@Pt-skin Pt_3_Ni CSFWs/C after 50,000 cycles.


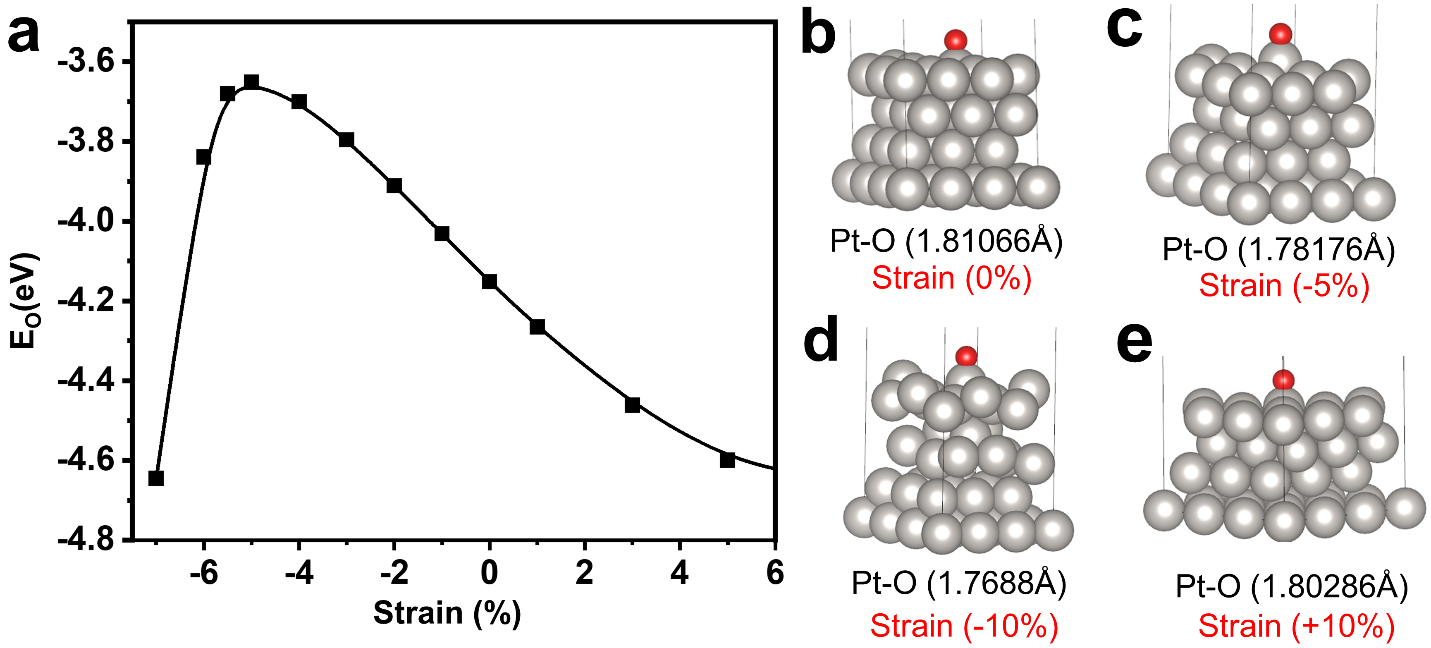
**Supplementary Figure 31.** DFT calculations of oxygen adsorption energy. (**a**) The relationship between the E_O_ on the fcc Pt (111) surface and strain varied from 6% (tensile) to -6% (compressive). (**b**-**e**) Atomic models of the Pt (111) surface with different strain. The gray and red spheres represent Pt and O atoms, respectively.


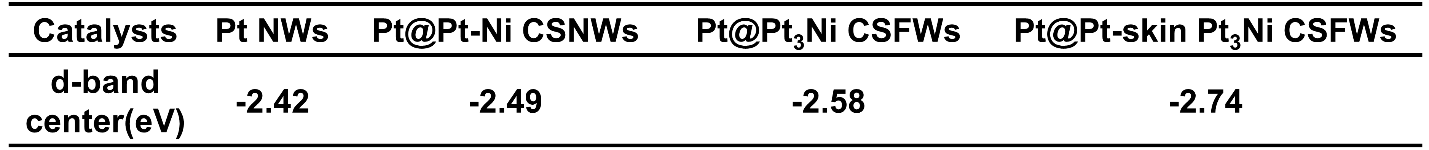
**Supplementary** **Table 1**. The d-band center of the samples calculated from high-resolution valence band UPS.


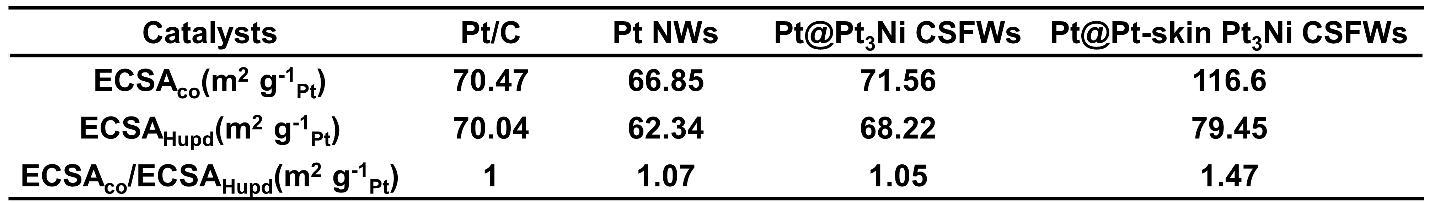
**Supplementary Table 2.** Comparison of ECSA_CO_ and ECSA_Hupd_ among the catalysts.


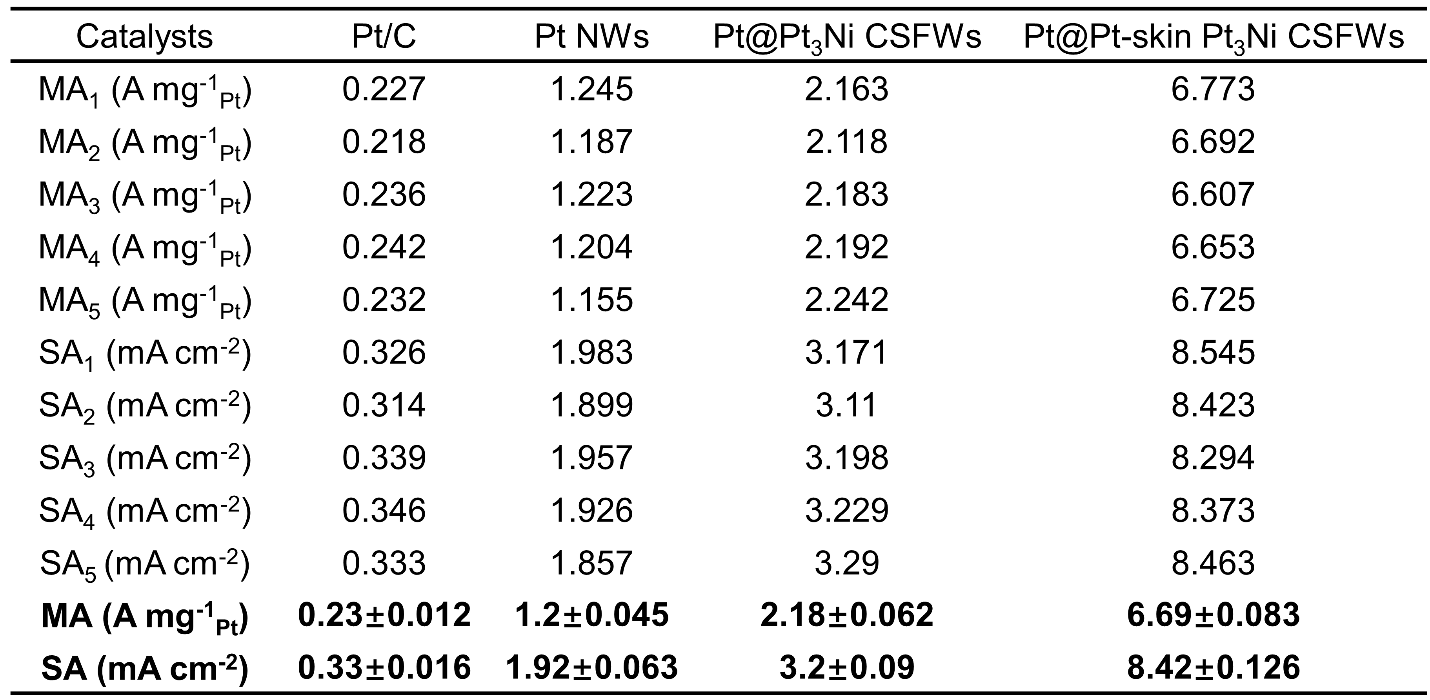
**Supplementary Table 3.** ORR activity comparison of Pt/C, Pt NWs, mesoporous Pt@Pt_3_Ni CSFWs and mesoporous Pt@Pt-skin Pt_3_Ni CSFWs based on 5 independent thin-film electrodes in 0.1 M HClO_4_ electrolyte.

**_1,2,3,4,5_:** The test number of mass activity and specific activity for each catalyst based on 5 independent thin-film electrodes.


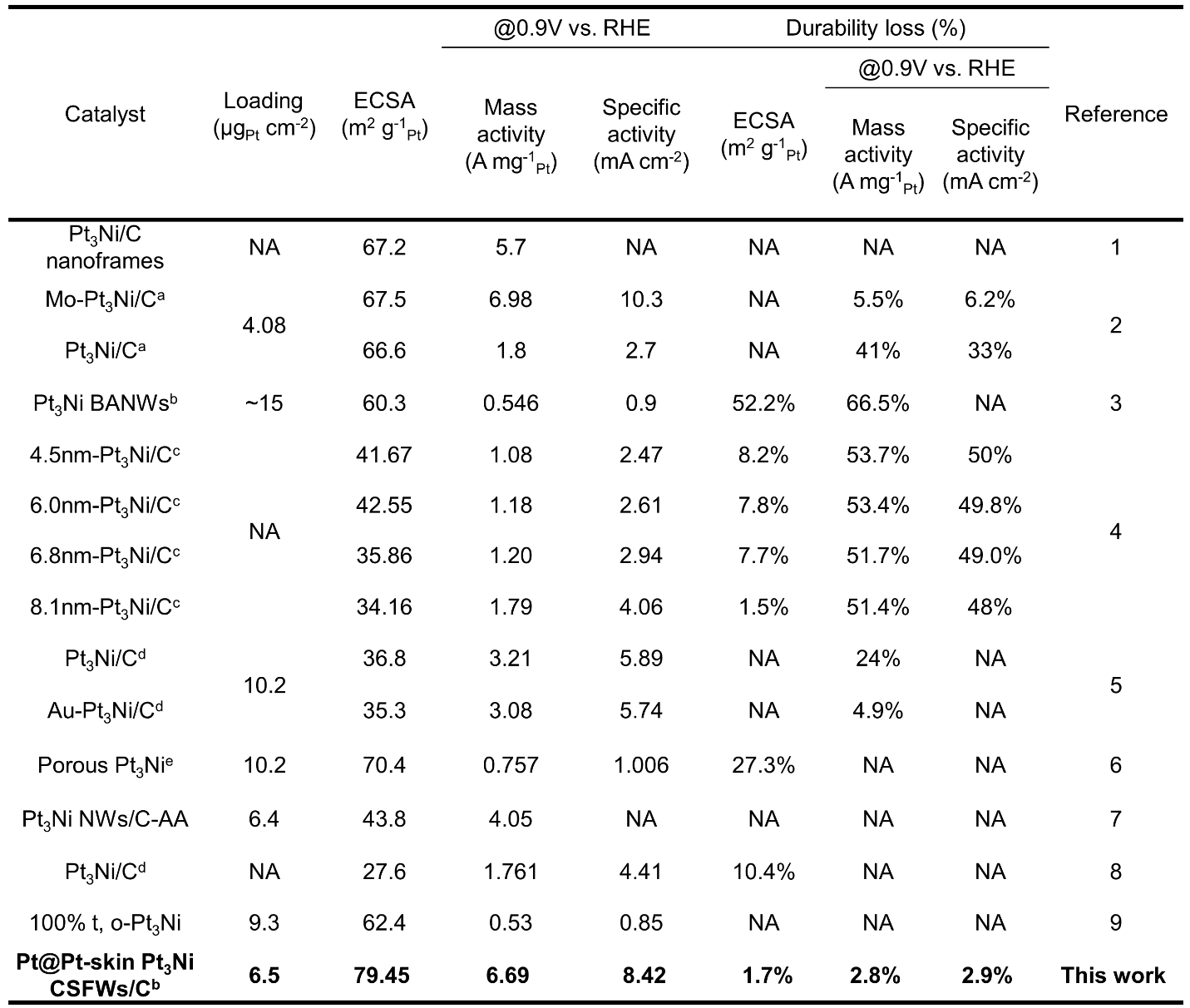
**Supplementary Table 4**. ORR activity comparison of our work with various Pt_3_Ni/C catalysts published in recent years.

NA: not available.

^a, b, c, d, e^: The durability loss of the catalysts is obtained after 8,000, 50,000, 4,000, 20,000 and 6,000 potential cycles, respectively.


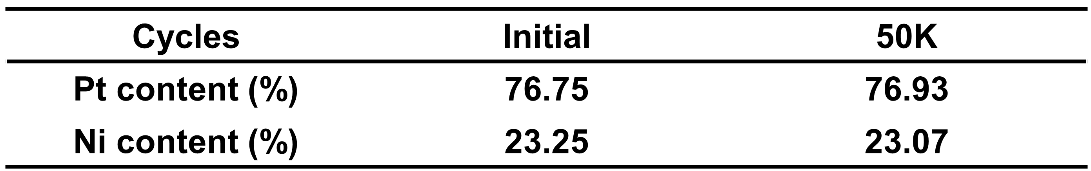
**Supplementary Table 5.** The Pt and Ni content of Pt@Pt-skin Pt_3_Ni CSFWs/C catalyst before and after potential-scanning cycles.


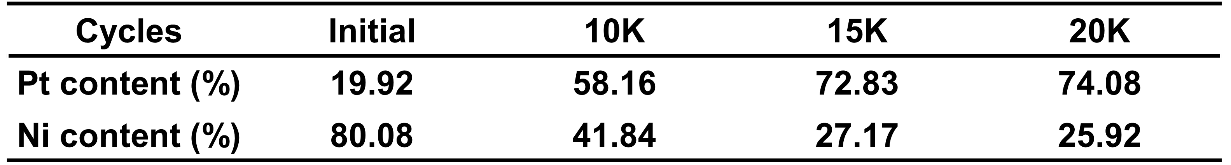
**Supplementary Table 6**. The Pt and Ni content of Pt@Pt-Ni alloy CSNWs/C catalyst before and after potential-scanning cycles.

Supplementary Reference

1. Chen, C. et al. Highly crystalline multimetallic nanoframes with three-dimensional electrocatalytic surfaces. *Science* **343**, 1339-1343 (2014).
2. Huang, X. et al. High-performance transition metal–doped Pt_3_Ni octahedra for oxygen reduction reaction. *Science* **348**, 1230-1234 (2015).
3. Gong, M. et al. One-Nanometer-Thick Pt_3_Ni Bimetallic Alloy Nanowires Advanced Oxygen Reduction Reaction: Integrating Multiple Advantages into One Catalyst. *ACS Catal.* **9**, 4488-4494 (2019).
4. Zhang, C., Hwang, S. Y. & Peng, Z. Size-dependent oxygen reduction property of octahedral Pt–Ni nanoparticle electrocatalysts. *J. Mater. Chem. A* **2,** 19778-19787 (2014).
5. Wu, Z. et al. Highly stable Pt_3_Ni nanowires tailored with trace Au for the oxygen reduction reaction. *J. Mater. Chem. A* **7,** 26402-26409 (2019).
6. Huang, X. et al. A facile strategy to Pt_3_Ni nanocrystals with highly porous features as an enhanced oxygen reduction reaction catalyst. *Adv. Mater.* **25**, 2974-2979 (2013).
7. Jiang, K. et al. Phase and Composition Tuning of 1D Platinum-Nickel Nanostructures for Highly Efficient Electrocatalysis. *Adv. Funct. Mater.* **27,** 1700830 (2017).
8. Zhu, J. et al. Active Pt_3_Ni (111) Surface of Pt_3_Ni Icosahedron for Oxygen Reduction. *ACS Appl. Mater. Interfaces* **8**, 30066-30071 (2016).
9. Wu, J. et al. Truncated octahedral Pt_3_Ni oxygen reduction reaction electrocatalysts. *J. Am. Chem. Soc.* **132**, 4984-4985 (2010).
